# Pioneer axons employ Cajal’s battering ram to enter the spinal cord

**DOI:** 10.1038/s41467-019-08421-9

**Published:** 2019-02-04

**Authors:** Ev L. Nichols, Cody J. Smith

**Affiliations:** 1 Department of Biological Sciences, University of Notre Dame, Notre Dame, IN 46556 USA; 2 Center for Stem Cells and Regenerative Medicine, University of Notre Dame, Notre Dame, IN 46556 USA

**Keywords:** Cellular neuroscience, Development of the nervous system, Glial development, Glial biology

## Abstract

Sensory axons must traverse a spinal cord glia limitans to connect the brain with the periphery. The fundamental mechanism of how these axons enter the spinal cord is still debatable; both Ramon y Cajal’s battering ram hypothesis and a boundary cap model have been proposed. To distinguish between these hypotheses, we visualized the entry of pioneer axons into the dorsal root entry zone (DREZ) with time-lapse imaging in zebrafish. Here, we identify that DRG pioneer axons enter the DREZ before the arrival of neural crest cells at the DREZ. Instead, actin-rich invadopodia in the pioneer axon are necessary and sufficient for DREZ entry. Using photoactivable Rac1, we demonstrate cell-autonomous functioning of invasive structures in pioneer axon spinal entry. Together these data support the model that actin-rich invasion structures dynamically drive pioneer axon entry into the spinal cord, indicating that distinct pioneer and secondary events occur at the DREZ.

## Abstract (web summary)

The fundamental mechanism of how sensory axons traverse a spinal cord glia limitans remains debatable, with some suggesting a role for boundary cap cells at the dorsal root entry zone (DREZ). Here, authors use time-lapse imaging of DRG axons at the DREZ to show that pioneer axons enter the DREZ before the presence of boundary cap cells, and that this entry is critically dependent on the development of actin-rich invasion structures reminiscent of invadopodia.

## Introduction

The somatosensory nerve detects sensory stimuli in the periphery and relays the information to the central nervous system (CNS)^1–3^. To ensure sensory information is rapid and uninterrupted, glial cells and axons coordinate at the dorsal root entry zone (DREZ), the CNS/peripheral nervous system (PNS) interface where sensory axons establish their dual domain nature^4,5^. During development, these sensory axons of the dorsal root ganglia (DRG) traverse into the spinal cord and glial cells reorganize^6,7^. This strict organization of the nerve is essential to drive somatosensory-induced behavior.

Our understanding of sensory nerve assembly and how numerous cell types dynamically interact during it are predicated on studies that have shown that ingrowth of sensory axons into the spinal cord occurs as neural crest cells are docked at the DREZ^7^. In contrast, Ramon y Cajal’s observation of pioneering 8th ganglion growth cones in developing otocyst presented a battering ram model where the growth cone employed an amoeboid mass to navigate through tissue with high cellular density^8^. Whether the battering ram is a distinct axonal structure or an overall term for the growth cone is unclear. Time-lapse imaging of pioneer axons shows that neural crest cells associate with the axons while they navigate to the DREZ, but the growth cone leads the neural crest cells^6^. In such imaging, however, the actual entry of the axons was not investigated. The temporal order of cellular assembly at the DREZ and whether there are distinct pioneer and secondary events, therefore, remains an unclear but critical question.

Classically, filopodia and lamellipodia have been described as the major motile structures in extending growth cones that aid navigation. Recently, other structures like invadopodia which are best understood in cancer cells as a method of cellular invasion and metastasis^9^ have been added to the repertoire of axon guidance structures^10^. These invadopodia impressively form in axons across phylogeny^10^. These data further demonstrate striking invadopodia morphology in growth cones and that invadopodia-related molecules are essential in motor axon navigation^10^; however, control of invadopodia in dynamic concert with classical growth cone machinery at specific anatomical decision points remains elusive. How the underlying dynamics drive growth cone machineries within distinct cellular milieu therefore remain unlinked and unexplored. These potential links could provide a step-wise blueprint for DREZ assembly.

Here, we used time-lapse imaging of pioneer axons in zebrafish to understand how pioneer sensory neurons dynamically grow into the spinal cord. We visualize the first axons crossing the glia limitans into the spinal cord. We first identify that neural crest boundary cells are absent from the DREZ during pioneer axon entry into the spinal cord. Without boundary cap cells to provide a substrate for ingrowth, actin-rich invasive structures, reminiscent of invadopodia, form in the growth cone. We then show invasion structures are essential for pioneer axons to enter the spinal cord. Using laser-induced lesions of the spinal tissue to mimic spinal cord breach, we demonstrate that we can bypass the necessity of invasive structures during axonal entry. Therefore, spinal ingrowth of the pioneer sensory axon is dependent on changing growth cone morphologies that invade into the spinal cord. We propose a modified model that invasive structures and boundary cap cells are both used by DRG axons but are temporally segregated by pioneer and secondary events.

## Results

### Neural crest cells are absent from DREZ during pioneer ingrowth

**Fig. 1:**
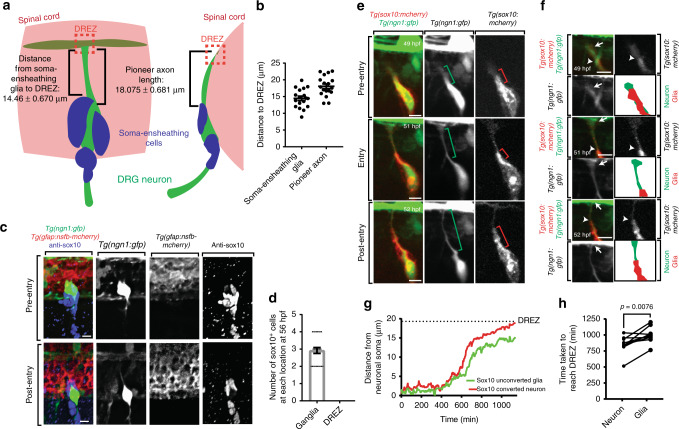
Sensory pioneer axons contact the dorsal root entry zone (DREZ) before arrival of boundary cap cells. **a** Diagram of the dorsal root ganglia (DRG) and DREZ following pioneer axon entry. Measurements are taken from (**b**). **b** Distance from the soma-ensheathing glia to the DREZ and length of the pioneer axon at 72 h post fertilization (hpf). **c** Confocal *z*-projection frames of a *Tg(ngn1:gfp); Tg(gfap:nsfb-mcherry)* zebrafish stained for Sox10 at 56 hpf showing absence of Sox10 staining at the DREZ. **d** Quantification of the number of Sox10^+^ cell bodies located in the DRG and at the DREZ at 56 hpf. SEM is shown, *n* = 25 DRG. **e** Confocal *z*-projection frames from a 24-h time-lapse starting at 48 hpf of a *Tg(sox10:gal4); Tg(uas:mcherry); Tg(ngn1:gfp)* animal before, during, and after pioneer axon entry. The mCherry^+^/GFP^-^ glia do not extend with the mCherry^+^/GFP^+^ neuron to the DREZ. Green brackets denote length of axon. Red brackets denote length of glia. **f** Growth cone insets and rendering from (**e**) confirming absence of glial processes at the DREZ. White arrows denote growth cone. White arrowheads denote glial leading edge. Green drawing denotes axon tracing. Red drawing denotes glial tracing. **g** Quantification of the distance between the pioneer axon (red) and associated glia (green) during axon extension, demonstrating DREZ contact by the pioneer axon before glial DREZ arrival. Dashed black line denotes the location of the DREZ. **h** Quantification of the time between axon initiation and axonal or glial contact of the DREZ. The axon consistently contacts the DREZ before associated glia, *n* = 9 DRG. Unpaired Student’s *t-*test (**h**). Scale bars denote 10 µm

Dorsal root ganglia neuronal ingrowth has been proposed to depend on neural crest cells that sit at the DREZ^7^. To test their role in DRG pioneer axon entry we used zebrafish to visualize their localization. At 48 h post fertilization (hpf), the DRG consists of a single neuron with a soma ensheathed by Sox10^+^ glia (Fig. 1a)^11^. Between 48 and 72 hpf, zebrafish dorsal root ganglia pioneer axons initiate dorsally, navigate to the DREZ, and cross into the spinal cord^6,11^. The DREZ is established 18.075 ± 0.681 µm (mean ± SEM) from the DRG neuronal soma (Fig. 1a, b). To test if boundary cap cells interact with the pioneer axon, we first used *Tg(ngn1:gfp)* animals to mark DRG neurons and stained for the neural crest cells with Sox10 at 56 hpf^12^. DRG development occurs in an anterior to posterior fashion. As a result, at 56 hpf the anterior-most DRG neurons (DRG ~1–10) have entered the spinal cord, the middle-located DRG (DRG ~11–20) are at or approaching the DREZ, and the posterior-most (DRG ~20–32) have not entered. This time point provides a developmental gradient of pioneer axon entry. In the ganglia, GFP^+^ neuronal cell bodies in both the anterior and posterior associated with ~3 Sox10^+^ cells. However, Sox10^+^ cell bodies were not present at the DREZ during these initial pioneer phases (Fig. 1c, d, *n* = 25 DRG). The localization of Sox10^+^ cells around the neuronal somas was evident when we measured the dorsal-most Sox10^+^ glia to the DREZ to be 14.46 ± 0.670 µm (Fig. 1a, b), consistent with soma-ensheathing glia^11,13^. These data, however, are inconsistent with the presence of a mature boundary cap population.

We next hypothesized that neural crest processes contact the DREZ before the pioneer axon^14^. To test this, we time-lapse imaged pioneer axon navigation in *Tg(ngn1:gfp); Tg(sox10:gal4); Tg(uas:mcherry)* animals by generating *z*-stacks of DRG every 5 min for 24 h starting at 48 hpf^15,16^. In these images, the pioneer axon appears yellow because of co-expression of mCherry and green fluorescent protein (GFP), and the glia appear red due to mCherry expression only. Initially, the pioneer axon and associated glia extend together (Fig. 1e, f). However, before the axon contacts the DREZ during entry the mCherry^+^ glia trail the growth cone, and when the axon reaches the DREZ, most of the axon is not associated with any glia (Fig. 1e, f). Following axon entry at the DREZ, glia continue navigation toward the DREZ (Fig. 1e, f). However, in these movies the glia only contacted the DREZ following axon spinal cord entry (Fig. 1e, f).

To further confirm this observation, we used *Tg(sox10:eos)* animals which express Eos protein in DRG cells^17^. With these animals, we could label single DRG cells by selectively exposing a specific 8 µm region to ultraviolet light, causing Eos fluorescent photoconversion^18^. At this stage in development, the DRG consists of only 3–4 cells (ensheathing glia and 1 neuron), allowing us to reliably photoconvert single DRG neurons before pioneer axon initiation while minimizing off-target effects^11,18^. After conversion, we created movies of pioneer axon navigation to the DREZ by collecting images every 5 min for 24 h. Cell tracings of these movies showed that the photoconverted pioneer axon contacted the DREZ before the non-photoconverted glia in 89% of our movies (Fig. 1g, Supplementary Figure 1a). Specifically, the pioneer axon contacts the DREZ 146.1 ± 41.21 min before glial DREZ contact (Fig. 1h, *n* = 9 DRG, *p* = 0.0076, paired Student’s *t-*test).

To rule out the possibility that thin, glial extensions that are not labeled with cytosolic markers contact the DREZ before neurons, we expressed membrane-tagged enhanced GFP (meGFP) under the *ngn1(−3.1* *kb)* promoter in *Tg(sox10:mrfp)* animals, which express membrane-tagged red fluorescent protein (mRFP) in all DRG cells^19^. In these animals, neuronal membranes are labeled with meGFP and mRFP to appear yellow, and glial membrane is labeled only with mRFP to appear red. Time-lapse images of DRG pioneer axons in these animals clearly show that yellow membranous processes from the pioneering growth cone contact the DREZ before the glial cell membrane (Supplementary Figure 1b). The totality of these complementary data are inconsistent with a model that pioneer axon ingrowth at the DREZ is dependent on neural crest cells.

### Pioneer axons change morphology during spinal cord entry

**Fig. 2:**
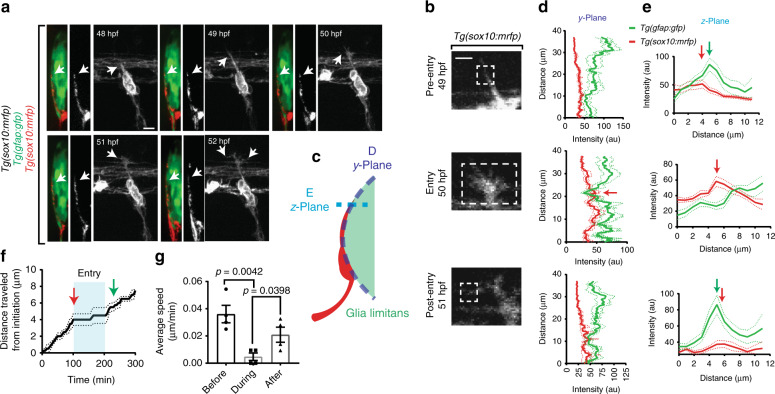
Pioneer axon changes morphology and velocity during glia limitans crossing. **a** Confocal *y*-orthoganol and *z*-projection frames from a 24-h time-lapse starting at 48 h post fertilization (hpf) of a *Tg(gfap:gfp); Tg(sox10:mrfp)* zebrafish showing axon navigation to the dorsal root entry zone (DREZ). White arrows denote the growth cone. **b** Deconvolved confocal single-frames from a 24-h time-lapse starting at 48 hpf of the pioneer axon of a *Tg(sox10:mrfp)* zebrafish before during and after glia limitans crossing. The growth cone swells during spinal entry. White box denotes the growth cone. **c**–**e** Diagram of the *y*- and *z*-planes of the DREZ showing how the graphs of (**d**, **e**) were taken on *y*-orthogonal images. Graphs in (**d**, **e**) denote intensity profiles of *Tg(gfap:gfp)* and *Tg(sox10:mrfp)* across the *y*- (**d**) and *z*-planes (**e**) of the growth cone in *y*-orthogonal images before, during, and after entry at the DREZ. The green fluorescent protein (GFP) intensity at the DREZ specifically decreases during axon entry. Red arrows denote decrease in *Tg(gfap:gfp)* intensity during pioneer axon entry. SEM is shown, *n* = 13 DREZ. **f** Quantification of the distance traveled by representative growth cones from the point of initiation through entry into the DREZ. Arrows denote time of axon (red) and glia (green) DREZ contact from Fig. 1g. Blue shaded box denotes the time the growth cone is at the DREZ. Blue lines denote SEM of 4 axons. **g** Average velocity form  of the growth cones before, during, and after entry. SEM is shown, *n* = 4 pioneer axons. Scale bars denote 10 µm in (**a**) and 5 µm in (**b**). Tukey’s honestly significant difference (HSD) (**g**)

Before the description of boundary cap cell populations, Ramon y Cajal described the appearance of amoeboid battering ram structures in developing axons^8^. To explore this possibility, we visualized the pioneer axon using *Tg(sox10:mrfp*) animals and radial glial cells that represent the glia limitans at the DREZ by expressing GFP with *gfap* regulatory sequences, *Tg(gfap:gfp)*^20^. At 48 hpf, the radial glial boundary separates the DRG cells from the spinal cord, and by 72 hpf, mRFP^+^ DRG axons have crossed the radial glia and formed anterior/posterior projections in the CNS (Fig. 2a). To understand how this cellular arrangement occurred, we expanded this analysis by visualizing the pioneer axon and radial glia every 5 min for 24 h during navigation into the CNS (Supplementary Figure 2a, Supplementary Movie 1). In these movies, the pioneer axon navigated along the radial glial boundary on the PNS side, then crossed the CNS/PNS radial glial boundary and formed anterior and posterior projections in the CNS (Fig. 2a). Before and after crossing of the glia limitans, the pioneer axon growth cone appears thin and finger-like (Fig. 2b). However, during entry the growth cone swells with a large amount of cytosol at its tip, reminiscent of Cajal’s description of a battering ram^21^ (Fig. 2b). Multiple growth cone swellings have been described including collapsed growth cones (in development), retraction bulbs, and dystrophic endings (in regeneration)^22,23^. We next sought to compare the growth cone structure at the DREZ to these structures using *Tg(sox10:mrfp)* animals. We visualized a collapsed growth cone as a DRG neurite retracted to the cell soma after it failed to specify as the single DRG axon. These images revealed a membranous flare at the growth cone tip, consistent with previous reports of retraction bulbs^23^ (Supplementary Figure 2a). We also tested if the growth cone structure at the DREZ was like a retraction bulb/dystrophic ending in a regenerative context by axotomizing spinal axons. Following injury, the spinal axon formed a spherical bulb lacking any projections (Supplementary Figure 2a), consistent with previous reports of retraction bulbs and dystrophic endings but distinct from the growth cone structures at the DREZ^22^. These data are consistent with the hypothesis that a unique growth cone structure is dynamically employed at the DREZ.

To confirm these morphological changes in the pioneer axon occurred during axonal crossing of the radial glial membrane, we quantified intensity profiles of *Tg(gfap:gfp)* and *Tg(sox10:mrfp)* along the glia limitans (*y*-axis) and through the growth cone (*z*-axis) as outlined in Fig. 2c. These intensity profiles demonstrated a decrease in *Tg(gfap:gfp)* intensity during entry (Fig. 2d, e). Following entry, the mRFP^+^ growth cone was localized medial to the glia limitans (Fig. 2d, e). As further confirmation of this entry event, we quantified the intensity of *Tg(gfap:gfp)* at the DREZ during the navigation of the *Tg(sox10:mrfp)*^*+*^ pioneer axons into the spinal cord. To do this we used tracking software to trace the intensity of GFP at the DREZ during entry. Single tracings of these data indicate that *Tg(gfap:gfp)* intensity at the DREZ decreases as the axon enters (Supplementary Figure 2b-d, *n* = 7 DRG, *p* = 0.0128). Dynamic *Tg(gfap:gfp)* intensity was also evident in other areas of the spinal cord but the *Tg(gfap:gfp)* intensity at the nascent DREZ was consistently decreased during pioneer axon spinal cord entry. To test this further, we measured the intensity of *Tg(gfap:gfp)* at the DREZ when pioneer axon entry into the spinal cord fails (as shown below). These measurements revealed no difference in *Tg(gfap:gfp)* intensity at the DREZ before and after failed axon entry into the spinal cord (Supplementary Figure 2e, f, *p* = 0.7742, *n* = 5 DREZ, Tukey’s HSD (honestly significant difference). Together, these data support the hypothesis that pioneer axons cross a radial glial membrane boundary by either penetrating that boundary or causing its rearrangement.

To understand the dynamics of this enlarged growth cone structure, we next traced the location of the pioneer growth cone during and after its navigation to the DREZ. These tracings revealed it formed during stalling of the pioneer axon at the DREZ (Fig. 2f, g, *n* = 4 axons). Following entry and disassembly of the growth cone structure at the DREZ, the axon sped back up, continuing its navigation.

To rule out that this stalling phenomenon is the pioneer axon waiting for glial arrival at the DREZ, we measured that stalling lasted at most 100 min following initial DREZ contact. Our data in Fig. 1e demonstrate that glial arrival at the DREZ occurs ~146 min after axon DREZ contact (Fig. 2g), arguing strongly against this hypothesis. Instead, these data are consistent with a model where the pioneer axon navigates dorsally using filopodia, stalls specifically at the DREZ to reorganize into a unique structure, and returns to filopodial navigation following entry.

### Actin-rich structures form at the DREZ

**Fig. 3:**
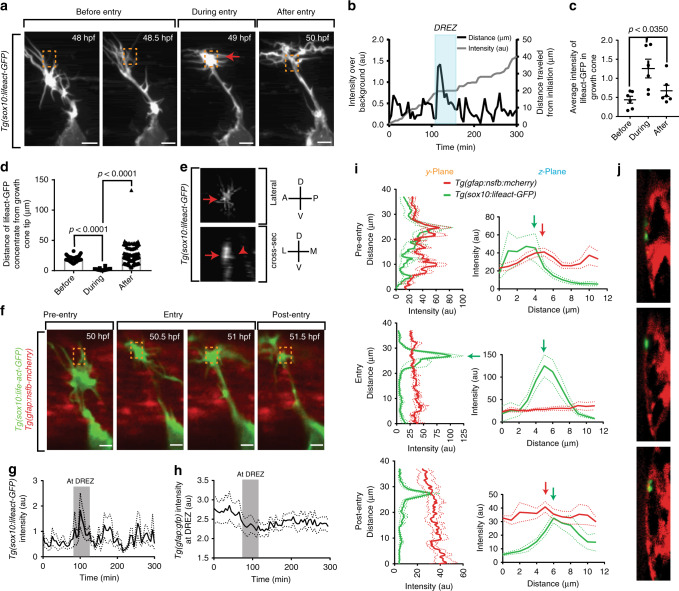
An actin-rich structure forms in pioneer axons during spinal entry. **a** Confocal *z*-projection frames from a 24-h time-lapse starting at 48 h post fertilization (hpf) of *Tg(sox10:lifeact-GFP*) zebrafish showing navigation of the pioneer axon into the spinal cord. Actin accumulates in the central growth cone specifically at the dorsal root entry zone (DREZ). Red arrows denote the actin-rich structure at the tip of the growth cone. Orange dotted box denotes DREZ. Entry occurs at 50 min. **b** Representative intensity profile of Lifeact-GFP at the growth cone (left *y*-axis, black line) and distance traveled by the growth cone (right *y*-axis, gray line). Actin concentration specifically occurs during stalling and entry. Shaded blue box denotes the period of axon entry at the DREZ. **c** Quantification of average intensity of Lifeact-GFP at the growth cone before, during, and after entry to the spinal cord. SEM is shown, *n* = 6. **d** Distance of Lifeact-GFP concentrate form the tip of the growth cone before, during, and after entry to the spinal cord. SEM is shown, *n* = 6. **e** Deconvolved confocal images of actin-rich structures in *Tg(sox10:lifeact-GFP)* animals in lateral and cross-sectional views demonstrating the basally projecting actin cluster at the DREZ. Red arrow denotes the same protruding actin concentrate. Red arrowhead denotes basally projecting lifeact-GFP protrusion. Compass shown to right of image denote directional locations on animal. D dorsal, V ventral, A anterior, P posterior, L lateral, M medial. **f** Confocal *z*-projection frames from a 24-h time-lapse starting at 48 hpf of *Tg(sox10:lifeact-GFP*); *Tg(gfap:nsfb-mcherry)* zebrafish showing navigation of the pioneer axon into the spinal cord. Orange dotted box denotes DREZ. **g** Intensity profile of Lifeact-GFP at the growth cone. Gray box denotes period of actin concentrate formation. SEM is shown, *n* = 11 pioneer growth cones. **h** Intensity profile of *Tg(gfap:nsfb-mcherry)* at the DREZ. Gray box denotes period of actin concentrate formation in the growth cone. mCherry intensity at the DREZ specifically decreases during actin accumulation in the growth cone. SEM is shown, *n* = 11 DREZ. **i** Intensity profiles of *Tg(sox10:lifeact-GFP)* and *Tg(gfap:nsfb-mcherry)* across the *y*- and *z*-planes of the growth cone in *y*-orthogonal images before, during, and after entry at the DREZ. The *y*- and *z*-planes determined as shown in Fig. 2b. White arrowhead denotes the glia limitans. Black arrowhead denotes the radial glia cell soma. SEM is shown, *n* = 11 DREZ. **j** The *y*-orthogonal images of used to create intensity profiles in (**i**). Scale bars denote 10 µm. Tukey’s honestly significant difference (HSD) (**d**)

In cancer, actin-rich invasion structures have been shown to be fundamentally important for cells to cross cellular boundaries^9^. Similar structures have also been recently shown to form in freely navigating axons grown in cell culture conditions and have been implicated in motor axon navigation^10^. We hypothesized that pioneer axon crossing into the spinal cord could utilize these invasive structures. We therefore visualized actin dynamics with Lifeact-GFP^24^, a marker for F-actin that does not perturb its dynamics, under *sox10* regulatory sequences, *Tg(sox10:gal4); Tg(uas:lifeact-gfp*)^24^. In time-lapse movies of pioneer axon navigation, the growth cone exhibited filopodia-like extensions before it reached the DREZ (Fig. 3a, Supplementary Movie 2). At the DREZ, Lifeact-GFP concentrated within the center of the growth cone, and most filopodial extensions retracted. This concentration of Lifeact-GFP dispersed as the axon returned to a filopodia-like morphology (Fig. 3a–c). The dynamic concentration of Lifeact-GFP corresponded with the swollen growth cone morphology at the DREZ. Intensity measurements of growth cone Lifeact-GFP show a distinct increase while at the DREZ (*n* = 6 DRG, *p* < 0.0350, Tukey’s HSD) (Fig. 3b, c). We next asked where in the growth cone the Lifeact-GFP concentration is present (e.g., tip of growth cone, center of growth cone, base of growth cone). To do this we quantified the distance of the actin concentration to the center of the growth cone. In this quantification, we measured that while at the DREZ, the Lifeact-GFP concentrate filled the growth cone center. Meanwhile, before and after entry the most intense concentration localized in the growth cone tips where the filopodia were present (*n* = 6 growth cones, *p* < 0.0001, Tukey’s HSD) (Fig. 3d). Actin-rich invasive structures have been shown to form basally projecting formations. We, therefore, used deconvolution software to determine if Lifeact-GFP concentrates were basally projecting. In this analysis we identified that while at the DREZ, Lifeact-GFP puncta exhibited thin basally projecting Lifeact-GFP structures (Fig. 3e). In contrast, the filopodia-like extensions that occurred before and after entry were not basally projecting. Together, these data support the hypothesis that pioneer axons dynamically reorganize their actin from filopodia into basally projecting concentrates specifically at the DREZ.

### Actin-rich structures cross the radial glia membrane

To test if these actin-rich structures are employed to cross the glia limitans, we time-lapse imaged actin in DRG neurons with Lifeact-GFP in an animal that also expressed mCherry in radial glia, *Tg(gfap:nsfb-mcherry)*^25,26^ (Fig. 3f, Supplementary Movie 3). Single planes of the CNS/PNS boundary revealed a reduction of mCherry intensity within radial glia during Lifeact-GFP rearrangement at the DREZ, suggesting that actin-rich structures could be utilized to cross the glia limitans (Fig. 3g, h, *n* = 8 DRG). We further tested this hypothesis with measurements of different axes as in Fig. 2c (Supplementary Movie 4). Before entry, Lifeact-GFP was present along a coherent *gfap*^*+*^ border in the *y*-plane and overlaid with the *gfap*^*+*^ peak in the *z*-plane before entry, suggesting that pioneer axons navigate along the radial glial boundary (Fig. 3i, j). During DREZ entry, the Lifeact-GFP intensity increases and the *gfap* intensity decreases to nearly zero (Fig. 3i, j). After the axon has entered, the *gfap*^*+*^ glia limitans peak is lateral to the Lifeact-GFP peak in the *z*-plane (Fig. 3i, j). These results are consistent with the hypothesis that actin-rich structures dynamically rearrange specifically to cross the glia limitans.

### Actin-rich structures form to cross a radial glial boundary

**Fig. 4:**
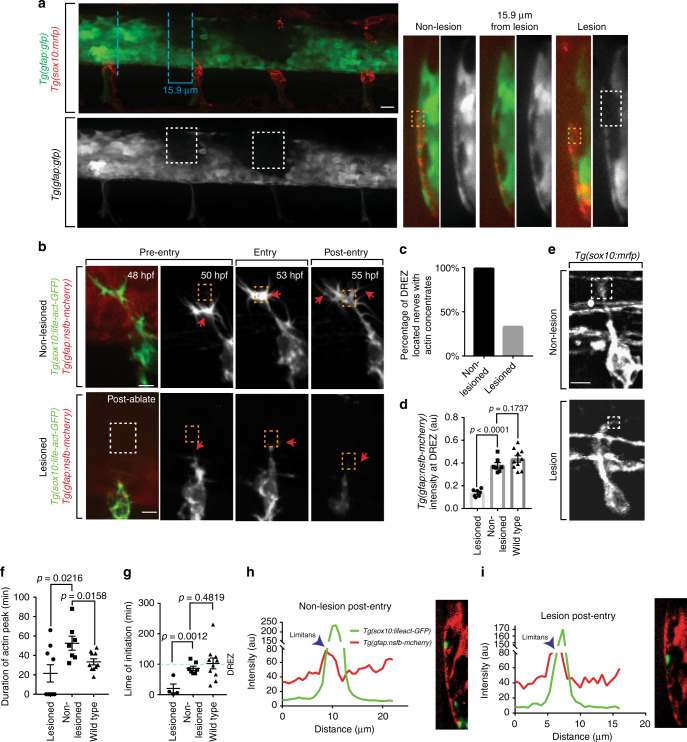
Actin-rich structures form to cross a radial glial boundary. **a** Confocal *z*-projection image of a *Tg(sox10:mrfp); Tg(gfap:gfp)* zebrafish after focal lesions at the dorsal root entry zone (DREZ), displaying the mechanism of focal ablations with adjacent, control non-lesioned nerves. Dashed blue lines indicate the plane shown in the *y*-orthogonal images. Dashed white boxes denote area of lesion. **b** Confocal *z*-projection frames from a 24-h time-lapse starting at 48 h post fertilization (hpf) of Lifeact-GFP, *Tg(gfap:nsfb-mcherry)* zebrafish showing navigation of the pioneer axon into the spinal cord in animals with a lesioned DREZ and a non-lesioned DREZ. Axons with lesioned DREZ fail to form actin concentrates. Red arrows denote the tip of the growth cone. White dotted box denotes the DREZ lesion. Orange dotted box denotes DREZ. **c** Quantification of DREZ-located axons that form actin-rich structures with a lesioned and non-lesioned DREZ. SEM is shown, *n* = 9 dorsal root ganglia (DRG) with a lesioned DREZ, *n* = 8 DRG with a non-lesioned DREZ. **d** Quantification of relative intensity of *Tg(gfap:nsfb-mcherry)* at the DREZ in wildtype (*n* = 10 DRG), lesioned (*n* = 9 DRG), and non-lesioned (*n* = 8 DRG) animals. SEM is shown. **e** Confocal images of a *Tg(sox10:mrfp)* growth cone during spinal cord entry with a lesioned and non-lesioned DREZ. **f** Quantification of duration of actin concentrates in wildtype, lesioned, and non-lesioned animals. SEM is shown, *n* = 10 wildtype animals, *n* = 8 non-lesioned animals, *n* = 9 lesioned animals. **g** Quantification of time of initiation of actin concentrates in wildtype, lesioned, and non-lesioned animals. Dotted blue line denotes when axons approach the DREZ. SEM is shown, *n* = 10 wildtype animals, *n* = 8 non-lesioned animals, *n* = 9 lesioned animals. **h** Representative intensity profile of *Tg(sox10:lifeact-GFP)* and *Tg(gfap:nsfb-mcherry)* through the *z*-plane of the growth cone and corresponding y-orthogonal image in a non-lesioned DREZ after entry. Measurements collected as described in Fig. 2b. **i** Representative intensity profile of *Tg(sox10:lifeact-GFP)* and *Tg(gfap:nsfb-mcherry)* through the *z*-plane of the growth cone and corresponding *y*-orthogonal image in a lesioned DREZ after axon entry. Measurements collected as described in Fig. 2b. Scale bars denote 10 µm. Tukey’s honestly significant difference (HSD) (**d**, **f**, **g**)

If actin-rich structures form to cross a radial glial membrane at the DREZ, then an incoherent DREZ boundary should render actin concentrates dispensable. To test this possibility, we created focal lesions in the radial glial at the DREZ. We first sought to test the feasibility of this paradigm using a diffraction-limited high-energy pulsed laser (Ablate©) to create a 12–14 µm size aberration in the spinal cord in *Tg(gfap:gfp); Tg(sox10:mrfp)* animals. Lesions were created at two DREZ per animal, and adjacent nerves in the imaging window were used as non-lesion controls (Fig. 4a). To confirm the specificity of the lesion, we digitally rotated these images 90 degrees across a single *y*-plane to visualize the lesion size at different spatial areas (Fig. 4a). In planes 15.9 µm away from the lesion, the glia limitans was indistinguishable from adjacent non-lesioned DREZ (Fig. 4a). To ensure that the lesion did not induce excessive damage to surrounding tissue we visualized overall tissue integrity with transmitted light. With transmitted light, damage surrounding the lesion was minimal (Supplementary Figure 3a, b). To rule out the possibility that the ablation only photobleached the glia limitans, we also visualized a loss of glial fibrillary acidic protein (GFAP) via immunohistochemistry (Supplementary Figure 3c). Next, we confirmed that axons approaching a lesioned DREZ were entering the spinal cord by digitally rotating the images of the axons after entry 90 degrees (Fig. 4a). In doing so, a clear mRFP^+^ intensity from the DRG axons is medial to the *gfap*^*+*^ boundary, supporting the conclusion that these axons entered the spinal cord (Fig. 4a).

We then created lesions in *Tg(sox10:lifeact-GFP); Tg(gfap:nsfb-mcherry)* animals at 48 hpf at the DREZ before the axons had reached the DREZ, then imaged *z*-stacks for 24 h (Fig. 4b, Supplementary Movie 5). In non-lesioned nerves, all pioneer axons produce actin-rich structures at the DREZ and enter the spinal cord (*n* = 8 DRG). In lesioned nerves, 33% of the pioneer axons produced actin-rich formations at the DREZ but all axons entered the spinal cord (*n* = 9 DRG) (Fig. 4c). We confirmed that the DREZ region was lesioned by scoring mCherry intensity and detected a decrease at lesioned DREZ (*n* = 8 non-lesioned, *n* = 9 lesioned, *p* < 0.0001, Tukey’s HSD) (Fig. 4d). We also observed no changes in the formation of anterior and posterior axonal projections inside the spinal cord across the treatment groups (Supplementary Figure 3d).

To characterize axon navigation in this paradigm, we traced pioneer axons during navigation in these movies and observed an absence of growth cone morphological changes as the axons approach the lesioned DREZ (Fig. 4e, Supplementary Figure 3e). Further, axons with lesioned DREZ displayed shorter actin concentrations (*n* = 9 lesioned, *n* = 8 non-lesioned, *p* = 0.0216, Tukey’s HSD) (Fig. 4f). In the few cases where actin concentrates did form, they initiated prematurely, indicating they entered in more ventral locations than the DREZ lesion (*n* = 9 lesioned, *n* = 8 non-lesioned, *p* = 0.0012, Tukey’s HSD) (Fig. 4g). We confirmed that pioneer axons entered the spinal cord in both the lesion and non-lesion conditions by digitally rotating images of the DRG and detecting the Lifeact-GFP^+^ axon inside the mCherry^+^ glia limitans (Fig. 4h, i). These data are consistent with the hypothesis that actin-rich structures form to breach a radial glial boundary and in the absence of such a boundary, actin-rich formations are dispensable.

### Perturbation of actin concentrates disrupts axon entry

**Fig. 5:**
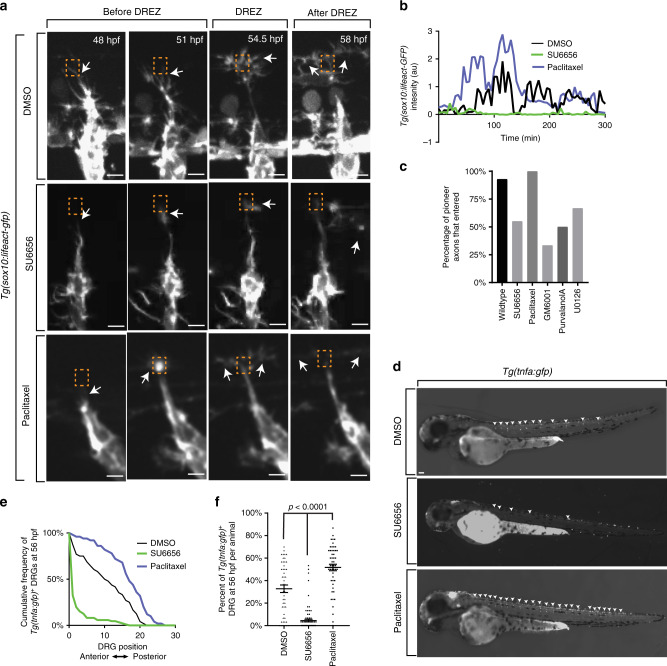
Actin-rich invasion components are necessary and sufficient for axon entry. **a** Confocal *z*-projection frames from a 24-h time-lapse starting at 48 h post fertilization (hpf) of Lifeact-GFP animals showing navigation of the pioneer axon into the spinal cord in animals treated with dimethyl sulfoxide (DMSO), SU6656, and paclitaxel. White arrows denote the tip of the growth cone. Orange dotted box denotes dorsal root entry zone (DREZ). Axon approaches DREZ at 300 min. **b** Representative intensity profiles of Lifeact-GFP expression at the growth cone in animals treated with DMSO (black), SU6656 (green), and paclitaxel (blue). SU6656-treated axons fail to form actin concentrates, while paclitaxel-treated axons form early and robust concentrates. Gray box denotes the formation of actin-rich invasion components in the DMSO- and paclitaxel-treated animals. **c** Quantification of axons that entered the spinal cord in animals treated with DMSO (*n* = 14 dorsal root ganglia (DRG)), SU6656 (*n* = 20 DRG), paclitaxel (*n* = 13 DRG), GM6001 (*n* = 18), purvalanol A (*n* = 18 DRG), and U0126 (*n* = 20 DRG). Inhibitors of cell invasion prevent axon entry. **d** Lateral images taken at 56 hpf of *Tg(tnfa:gfp)* animals treated with DMSO, SU6656, or/and paclitaxel. Arrowheads denote *Tg(tnfa:gfp)*^*+*^ DRG. **e** Cumulative frequency of animals treated with SU6656, paclitaxel, and DMSO that express *Tg(tnfa:gfp)* at each DRG at 56 hpf; *n* = 86 SU6656-treated fish, *n* = 50 paclitaxel-treated fish, *n* = 48 DMSO-treated fish. **f** Bar graph of the percentage of DRG that were *Tg(tnfa:gfp)*^+^ at 56 hpf in animals treated with SU6656, paclitaxel, and DMSO; *n* = 86 SU6656-treated fish, *n* = 50 paclitaxel-treated fish, *n* = 48 DMSO-treated fish. SEM is shown. Scale bars denote 10 µm in (**a**) and 0.1 mm in (**d**). One-way analysis of variance (ANOVA) (**f**)

If actin-rich structures are employed in the growth cone to cross the glia limitans, then disrupting them should perturb entry of axons into the spinal cord. The requirement of invasion components in precursor events to DREZ formation prevented the use of genetic mutants to test their requirement at the DREZ^27^. Therefore, we first tested bioactive molecules shown to alter actin-rich invasion structures. We identified two molecules that had strong opposite effects on the actin-rich structures that form in pioneer axons at the DREZ^9,10,27,28^. SU6656 disrupted the formation of actin concentrates in pioneer axons (Fig. 5a, b, Supplementary Figure 4a). SU6656 inhibits Src family kinases which promote the formation of invadopodia, an important mechanism of cancer cell invasion^9,28^. SU6656 is highly selective for Src kinases compared to other protein kinases^29^. Paclitaxel, which stabilizes microtubules by direct binding and has been shown to promote invadopodia formation, increased the duration of actin concentrates in pioneer axons (Fig. 5a, b)^28^. Differences in the morphologies of DRG and their axons were indiscernible across treatments (*n* = 14 dimethyl sulfoxide (DMSO); *n* = 20 SU6656; *n* = 13 paclitaxel) (Supplementary Figure 4b-d). Similarly, no significant defects in gross morphology of treated animals were detected (Supplementary Figure 4e).

We next tested if treatment of SU6656 perturbs actin-rich invasion component formation. To do this we treated animals with SU6656 at 36–72 hpf, after neural crest migration is completed^17^. We then imaged *z*-stacks of *Tg(sox10:lifeact-GFP*) animals every 5 min for 24 h starting at 48 hpf (Fig. 5a, Supplementary Movie 6). Measurements of Lifeact-GFP peak during navigation revealed an abrogation of the actin peak (Fig. 5b, *n* = 10 DMSO-treated, *n* = 14 SU6656-treated), and only 60% of SU6656-treated axons produced actin concentrates and 55% entered the spinal cord (*n* = 20 DRG) (Fig. 5c). All SU6656-treated axons that entered the spinal cord formed actin concentrates, supporting the hypothesis that invasion components are essential to enter the spinal cord (*n* = 20 DRG) (Fig. 5c). As confirmation that Src inhibition reduces stability of invasive structures, we scored that SU6656-treated pioneer axons formed actin peaks later than control animals (*n* = 14 treated DRG, *n* = 14 untreated DRG, *p* = 0.0298, Tukey’s HSD) (Supplementary Figures 4f-h).

To further test if invasion components are required to cross the DREZ, we tested additional bioactive molecules that disrupt cell invasion^28^. We identified that Cdk inhibitor Purvalanol A, MEK inhibitor U0126, and pan-MMP inhibitor GM6001 also disrupt axon entry. Treatment with these drugs at 36 hpf phenocopied entry deficits of SU6656 treatment (Fig. 5c, Supplementary Figure 4i). We did not visualize differences in DRG number, axon initiation defects, or deficiencies in navigation to the DREZ in these treatments (Supplementary Figures 4b-d). It should be noted that GM6001 can also inhibit other zinc-dependent proteinases and U0126 has been shown to prevent calcium transients in some cells^30,31^. Purvalanol A is specific to Cdk family members^32^. Taken together, these complementary data support the hypothesis that actin-rich invasion components are required to enter at the DREZ.

### Invasion components are sufficient for spinal cord entry

If invasion components are sufficient to cross the radial glia boundary at the spinal cord, then their formation before axon arrival to the DREZ should result in premature axon entry. To test this, we treated *Tg(sox10:lifeact-GFP)* animals at 36 hpf with paclitaxel, a molecule known to stabilize invadopodia (Fig. 5b)^28^ and time-lapse imaged those animals for 24 h starting at 48 hpf (Fig. 5a, Supplementary Movie 7). To confirm that paclitaxel impacts actin-rich invasion components in DRG pioneer axons, we measured Lifeact-GFP intensities in animals treated with paclitaxel and quantified an increase in the duration of actin concentrates (*n* = 13 paclitaxel-treated, *n* = 14 DMSO-treated, *p* = 0.0001, Tukey’s HSD) (Supplementary Figure 4h). These invasion components also formed earlier in the axon’s navigation (Fig. 5b, *n* = 10 DMSO-treated, *n* = 14 paclitaxel-treated, Supplementary Figure 4f, g). In addition, animals treated with paclitaxel displayed shorter axons that entered early (*n* = 42 paclitaxel, *n* = 16 DMSO, *p* = 0.0270, Student’s *t*-test) and larger axonal angles as measured from the point of axon bifurcation to the DRG soma (*n* = 42 paclitaxel, *n* = 15 DMSO, *p* = 0.0004, Student’s *t*-test) (Supplementary Figure 4j, k). These results are consistent with the hypothesis that paclitaxel-treated axons induce invasive structures and enter the spinal cord prematurely.

We also validated axon entry with a second, complementary approach. Tumor necrosis factor-ɑ (TNFɑ) is upregulated at pioneer axons as they enter the DREZ^6^. We treated *Tg(tnfa:gfp)* animals with SU6656, paclitaxel, or DMSO at 36 hpf and quantified the number of *Tg(tnfa:gfp)*^*+*^ DRG at 56 hpf to visualize a temporal gradient of DRG entry. If paclitaxel causes premature entry, then paclitaxel-treated animals should have increased numbers of *Tg(tnfa:gfp)*^*+*^ DRG compared to DMSO-treated animals in posterior DRG. Our results showed that paclitaxel treatment resulted in an average of 15.5 *Tg(tnfa:gfp)*^*+*^ DRG (*n* = 50 animals), compared to 9.83 in DMSO controls (*n* = 48 animals) and 1.35 in SU6656-treated animals at 56 hpf (*n* = 86 animals) (Fig. 5d–f). Consistent with the hypothesis that paclitaxel and SU6656 effect spinal invasion and entry, whole animal profiles of *Tg(tnfa:gfp)*^*+*^ DRG showed a loss of anterior *Tg(tnfa:gfp)*^*+*^ DRG in SU6656-treated animals compared to DMSO. Meanwhile, an expansion of posterior *Tg(tnfa:gfp)*^*+*^ DRG in paclitaxel-treated animals was observed compared to DMSO (Fig. 5f). We ensured that the observed *tnfa* expression was in the DRG by treating *Tg(tnfa:gfp); Tg(sox10:mrfp)* with SU6656 or paclitaxel. In both DMSO-treated and paclitaxel-treated animals by 72 hpf, 100% of pioneer neurons express GFP in DMSO-treated compared to 51.7% of SU6656-treated pioneer neurons (*n* = 60 DMSO-treated, *n* = 68 paclitaxel-treated, *n* = 118 SU6656-treated) (Supplementary Figure 4l, m). These results support the hypothesis that precise regulation of invasion components at the DREZ drives axon entry.

### Invasion structures in pioneer axons are required for entry

**Fig. 6:**
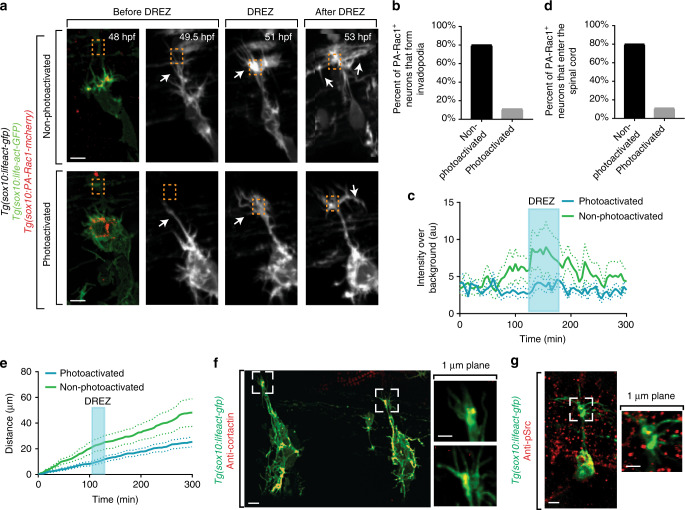
Invadopodia are cell autonomously required for spinal entry. **a** Confocal *z*-projection images taken from a 24-h time-lapse starting at 48 h post fertilization (hpf) of a *Tg(sox10:lifeact-GFP); Tg(sox10:PA-Rac1-mcherry)* zebrafish with both photoactivated and non-photoactivated Rac1. Orange box denotes dorsal root entry zone (DREZ). White arrow denotes growth cone. **b** Quantification of the number of PA-Rac1^+^ neurons that enter the spinal cord. Photoconverted neurons fail to enter the spinal cord. SEM is shown, *n* = 10 non-photoactivated dorsal root ganglia (DRG), *n* = 9 photoactivated DRG. **c** Quantification of the number of PA-Rac1^+^ neurons that form actin-invasive structures. Photoactivated neurons fail to form actin-invasive components. SEM is shown, *n* = 10 DRG. **d** Intensity profiles of growth cone Lifeact-GFP in neurons with photoactivated (*n* = 9) and non-photoactivated (*n* = 10) Rac-1. **e** Distance traveled by neurons with photoactivated (*n* = 9) and non-photoactivated (*n* = 10) Rac-1. **f** Confocal of two growth cones of a *Tg(sox10:lifeact-GFP)* animals stained for Cortactin showing co-localization of Cortactin with Lifeact-GFP foci at the DREZ. **g** Confocal images of a *Tg(ngn1:gfp)* animals stained for pSrc showing localization of pSrc in pioneer growth cones. Scale bars denote 10 µm in (**a**) and 5 µm in (**f**, **g**). Insets in (**f**, **g**) are single-plane confocal images

We next tested the hypothesis that actin-rich invasion structures were specifically required in the growth cones of pioneer axons. Rac1 drives disassembly of invadopodia, an invasive structure that mimics our Lifeact-GFP concentrates^33^. We therefore manipulated Rac1 specifically in pioneer axons using a photoactivable Rac1. We expressed PA-Rac1 in pioneer axons by injecting *uas:PA-Rac1-mcherry* into *Tg(sox10:gal4); Tg(uas:lifeact-gfp)*. We confirmed the expression of PA-Rac1-mcherry by detecting mCherry^+^ puncta in pioneer axons (Fig. 6a). Rac1 was photoactivated during DREZ entry and navigation by exposing the tissue to 445 nm light every 5 min for 24 h. As a control, PA-Rac1 expressing neurons were imaged without exposure to 445 nm. In this experiment, 80% of unactivated PA-Rac1 neurons produced Lifeact-GFP concentrates compared to 10% of activated PA-Rac1 neurons (Fig. 6b, c, *n* = 9 unactivated, *n* = 10 activated). Similarly, 10% of activated PA-Rac1 axons entered the spinal cord compared to 80% of unactivated controls (Fig. 6d, *n* = 9 unactivated, *n* = 10 activated). Activated PA-Rac1 DRG still stall at the DREZ but fail to form stable Lifeact-GFP structures (Fig. 6c, e). Meanwhile, unactivated controls stall and form actin concentrates normally. Together, these data support the hypothesis that invasive structures are cell autonomously required in pioneering growth cones for spinal cord entry.

### Invasion components colocalize with invadopodia indicators

Since the actin-rich invasion components in pioneer axons respond to pharmacological treatments and molecular triggers that alter invadopodia, we used immunohistochemistry to test the localization of invadopodia molecules during axon entry. Invadopodia recruit Cortactin and a phosphorylated form of Src to drive their formation^9^. We stained *Tg(sox10:lifeact-gfp)* animals at 56 hpf to visualize localization of these components with actin concentrates at the DREZ. Cortactin localized specifically at the growth cone at the DREZ (Fig. 6f). Using deconvolution software we visualized that this punctate pattern of Cortactin colocalized with Lifeact-GFP puncta that were basally projecting (Fig. 6f). pSrc also colocalized to the growth cone at the DREZ (Fig. 6g). Together, these data are consistent with the hypothesis that the invasion components utilized during axon entry are invadopodia^9,10,34^.

### Invadopodia defects can be rescued with spinal lesions

**Fig. 7:**
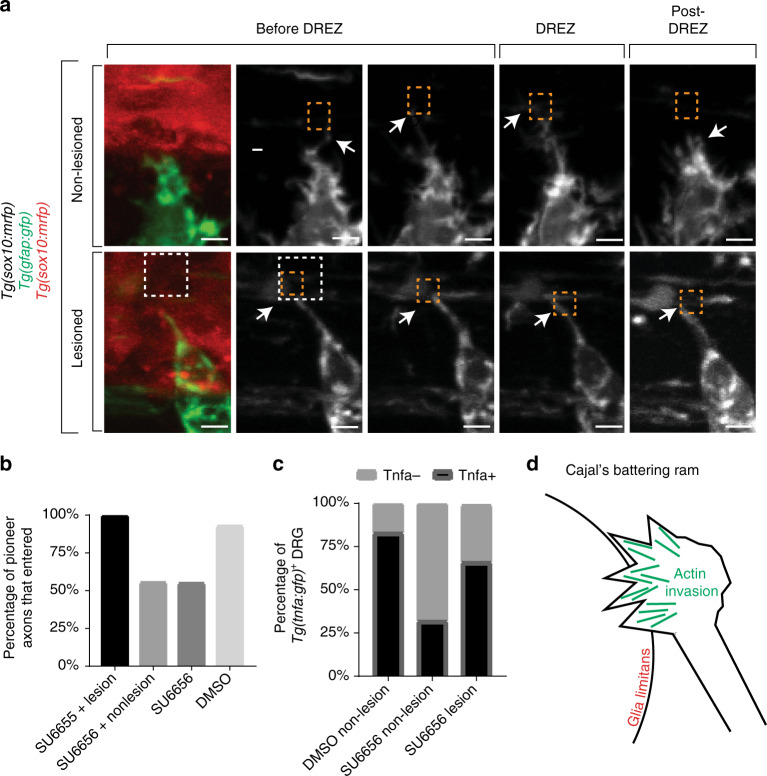
Entry defects from failed invasion are rescued by dorsal root entry zone (DREZ) lesioning. **a** Confocal *z*-projection frames from a 24-h time-lapse starting at 48 h post fertilization (hpf) of *Tg(gfap:gfp), Tg(sox10:lifeact-gfp)* zebrafish treated with SU6656 with and without a DREZ lesion showing pioneer axon navigation. **b** Quantification of pioneer axon entry in animals treated with SU6656 and a lesioned DREZ (*n* = 12), SU6656 without a lesioned DREZ (*n* = 9), SU6656 (*n* = 20), and dimethyl sulfoxide (DMSO; *n* = 14). SEM is shown. **c** Quantification of *Tg(tnfa:gfp)*^+^ dorsal root ganglia (DRG) in animals treated with DMSO without a DREZ lesion (*n* = 30), animals treated with SU6656 without a DREZ lesion (*n* = 30), and animals treated with SU6656 with a DREZ lesion (*n* = 28). **d** Schematic representation of actin-rich invasion that comprises Cajal’s battering ram. Scale bars denote 10 µm

Our data thus far indicate that actin-based invasion is dynamically employed to breach the glia limitans. This implies that pioneer axons could enter the spinal cord despite invadopodia inhibition if a coherent glia limitans is absent. To test this we asked if DREZ lesioning could rescue axon entry in SU6656-treated animals. We did this by treating *Tg(sox10:lifeact-GFP); Tg(gfap:nsfb-mcherry)* animals with SU6656 or DMSO at 36 hpf, lesioning the DREZ at 48 hpf, and then scored navigation and invadopodia formation in pioneer axons from 48 to 72 hpf (Fig. 7a, Supplementary Movie 8). In SU6656-treated animals with lesioned DREZ the Lifeact-GFP peak was shorter, consistent with SU6656 treatment (Supplementary Figure 5a-d). We confirmed the lesion by measuring a difference in the *Tg(gfap:nsfb-mcherry)* intensity in lesioned and non-lesioned DREZ (Supplementary Figure 5e). In SU6656-treated non-lesioned DREZ, pioneer axons rarely entered the spinal cord. However, SU6656-treated animals with lesioned DREZ fully recovered spinal cord entry (Fig. 7b). These data are consistent with the hypothesis that invadopodia are dispensable for pioneer axon entry when an intact DREZ is absent.

We next used a complementary approach of *Tg(tnfa:gfp)* expression to test the above hypothesis. To test this we treated *Tg(tnfa:gfp); Tg(gfap:nsfb-mcherry)* animals with SU6656 or DMSO at 36 hpf, lesioned the DREZ at 48 hpf, and then scored *Tg(tnfa:gfp)* expression in pioneer axons at 72 hpf. In SU6656-treated animals with lesioned DREZ, 65% of DRG neurons expressed *Tg(tnfa:gfp)*, a rescue from SU6656-treated non-lesioned DREZ (Fig. 7c). Based on these collective data, we propose that invadopodia induce axon entry into the spinal cord and crossing of the glia limitans in the absence of boundary cap cells (Fig. 7d).

## Discussion

Somatosensory nerve development requires the coordination of neuronal and glial cells. Using time-lapse imaging we first demonstrate that boundary cap cells are not present at the DREZ during pioneer axon entry, altering our understanding of how DRG axons traverse at the nascent DREZ. Instead, invadopodia dynamically form in a distinct growth cone structure when pioneer axons approach the DREZ and are essential for axons to breach the spinal cord glia limitans. Using laser-induced lesions of spinal tissue we show that failed invadopodia formation and axon entry can be rescued by creating an artificial breach of the glia limitans at the DREZ. Together, the simplest explanation for these combined data is that pioneer axons employ a unique growth cone structure at minimum composed of invadopodia to cross the spinal cord boundary.

Boundary cap cells are neural crest-derived cells that reside at both the DREZ and motor exit point^7,35–37^. Some reports have observed that axons preferentially cross into the spinal cord at the sites of boundary cap cells, leading to the hypothesis that boundary cap cells guide DRG axon ingrowth^7^. Based on the collective data we propose, the invasion and boundary cap ingrowth hypotheses are both likely but separated temporally in development: pioneer axons utilize invadopodia to enter and secondary axons enter via neural crest cells that reside at or near the DREZ^7^.

Axon navigation through space utilizes distinct and dynamic growth cone morphologies^8,10,38–41^. In addition to the classical growth cone structures of filopodia and lamellipodia, invadopodia have recently been added to the repertoire of growth cone machineries^10^. Specifically, invadopodia are required during early motor axon navigation, providing a potential link between axon guidance studies and invasion through packed tissues. We identify a precise time in development where a growth cone switches morphology within ~100 min: filopodia before entry, to invadopodia during entry, and back to filopodia following entry. Our data suggest that the DREZ could serve as a model to study the interaction between guidance cues and growth cone morphologies, linking two well-studied fields: axon guidance and cell invasion.

Axonal invadopodia colocalize with Cortactin and pSrc. Further, we modulate the invasive capacity of pioneer axons by inhibiting Src family kinases. We also identified Rac1 as a negative regulator of invasion components in DRG pioneer axons. Together, these results are consistent with reports of invadopodia formation in other contexts, including growth cones in vitro^9,10,27,42–44^. Invadopodia are essential in at least three distinct neurodevelopmental times: neural crest migration^27^, motor axon navigation^10^, and pioneer axon entry. Evidence suggesting the reuse of these components is particularly intriguing since defects could disrupt multiple stages of circuit formation.

## Methods

### Contact for reagent and resource sharing

Further information and requests for resources and reagents should be directed to the Lead Contact, Cody J. Smith (csmith67@nd.edu).

### Experimental model and subject details

All animal studies were approved by and complied with the University of Notre Dame Institutional Animal Care and Use Committee. Stable zebrafish strains used for this study were as follows: AB, *Tg(ngn1:gfp)*^12^*, Tg(sox10:mrfp)*^19^*, Tg(gfap:gfp)*^20^*, Tg(sox10:gal4)*^15^*, Tg(uas:lifeact-gfp)*^24^*, Tg(gfap:nsfb-mcherry)*^25^*, Tg(tnfa:gfp)*^45^, and *Tg(sox10:eos)*^17^, *Tg(uas:mcherry)*^16^. A transgenic *Tg(sox10:mrfp); ngn1:egfp-caax* was generated by injecting a *ngn1:egfp-caax* construct into *Tg(sox10:mrfp)* embryos at a 75 ng/µl concentration and Tol2 396 transposase messenger RNA (mRNA). F0 embryos were imaged for mosaic analysis. This construct was made using the −3.1 kb *ngn1* promoter^46^.

Embryos were produced from pairwise matings and raised at 28 °C in egg water in constant darkness and staged by hours or days post fertilization^47^. Embryos of either sex were used for all experiments.

### In vivo imaging

Embryos were manually dechorionated at 48 hpf and anesthetized with 3-amino-benzoic acid ester (Tricaine). Anesthetized embryos were immersed in 0.8% low-melting point agarose and mounted on their right side in glass-bottomed 35 mm Petri dishes^48^. A spinning disk confocal microscope from 3i technology^©^ was utilized and is equipped with a Zeiss Axio Observer Z1 Advanced Mariana Microscope with X-cite 120LED White Light LED System and filter cubes for GFP and mRFP, a motorized X,Y stage, piezo Z stage, 20X Air (0.50 NA) objective with 2 mm working distance, 63× (1.15 NA) water objective with 0.66 mm working distance, 40× (1.1 NA) water objective with 0.62 mm working distance, CSU-W1 T2 Spinning Disk Confocal Head (50 μM) with 1× camera adapter, and/or iXon3 1Kx1K EMCCD camera, dichroic mirrors for 446, 515, 561, 405, 488, 561, 640 excitation, laser stack containing 405 nm, 445 nm, 488 nm, 561 nm, and 637 nm with laser stack FiberSwitcher that has 250 μs switch time, photomanipulation with vector^©^ high speed point scanner ablations at diffraction limited capacity, Ablate!TM^©^ Photoablation System (532 nm pulsed laser, pulse energy 60J @ 200 HZ). All images of DRG were taken in the trunk of the animal. Time lapse images were taken every 5 min for 24 h starting at 48 hpf. Adobe Illustrator and ImageJ were used to process images and enhance image brightness and contrast.

### Radial glia focal lesions

Focal lesions of the radial glia membrane were created using the Ablate!TM^©^ Photoablation System described above. Two lesions of 10–12 µm were created approximately 10 µm from the bottom of the radial glial membrane (the location of the DREZ) in each animal at 48 hpf. Adjacent nerves with non-lesioned DREZ areas were used as controls. After focal lesioning, time-lapse images of the nerves were taken as described above.

### Immunohistochemistry

The primary antibodies used in this study were Zrf-1 (1:100, mouse, ZIRC, ZFIN ID: ZDB-ATB-081002–46), anti-Sox10 (1:5000, rabbit, Kucenas lab)^49^, anti-Cortactin (1:200, mouse, Sigma, catalog number: SAB4500766)^10^, and anti-pSrc (Y418) (1:200, rabbit, Invitrogen, catalog number: 44–660G)^10^. The secondary antibodies used were Alexa Flour 594 goat anti-rabbit (1:600, Invitrogen, catalog number: A-11037), Alexa Flour 594 goat anti-mouse (1:600, Invitrogen, catalog number: R37121), and Alexa Fluor 647 goat anti-mouse (1:600, Invitrogen, catalog number: A-21235). Larvae were fixed using 4% paraformaldehyde in PBST (phosphate-buffered saline (PBS) with 0.1% Triton X-100) at room temperature (25 °C) for 3 h. The fixed larvae were washed with PBST, deionized water with 1% Triton X-100, and acetone for 5 min each, and incubated in acetone at −30 °C for 15 min. Larvae were washed 3 times with PBST for 5 min and incubated with 5% goat serum in PBST for 1 h at room temperature. The larvae were then incubated with the primary antibody solution for 1 h at room temperature and then transferred to 4 °C overnight. After 3 washes with PBST for 30 min and a wash with PBST for 1 h, the larvae were incubated with the secondary antibody solution for 1 h at room temperature and then transferred to 4 °C overnight. After 3 washes in PBST for 1 h, larvae were stored in 50% glycerol in PBS at 4 °C until imaging. Larvae were mounted and still images were taken using the same protocol as previously described for in vivo imaging.

### Chemical treatments

The chemical reagents used for this study were SU6656 (Santa Cruz Biotechnology), paclitaxel (Acros), GM6001 (Santa Cruz Biotechnology), purvalanol A (Tocris), and U0126 (Cayman). Stock solutions of these reagents were kept at −20 °C with concentrations of 375 µM (SU6656), 2.2 mM (paclitaxel), and 1.0 mM (GM6001, purvalanol A and U0126) in DMSO. Treated embryos were dechorionated at 36 hpf and incubated with 3 µM (SU6656)^27^, 22 µM (paclitaxel)^50^, and 10 µM (GM6001, purvalanol A and U0126) in egg water until imaging^28^. Control animals were incubated with 1% DMSO in egg water.

### Intravital zebrafish imaging

*Tg(tnfa:gfp)* animals were incubated with SU6656, paclitaxel, or DMSO and imaged using a Zeiss Axiozoom microscope equipped with 4 filter cubes (4′,6-diamidino-2-phenylindole (DAPI), GFP, RFP, and 641 nm) and a monochrome camera run by Zen software at 56 hpf. The number of *Tg(tnfa:gfp)*^+^ DRG were scored for each treatment. Images were taken with a monochrome camera run by Zen software. Images were processed using Adobe Photoshop to adjust brightness and contrast.

### Molecular biology

Creation of the psELN07 (*ngn1:megfp:pa+cmcl2:gfp)* DNA construct was generated using the Gateway LR Clonease II Plus system (ThermoFisher) using zebrafish compatible Tol2 vectors^51^. A *p5e-ngn1(−3.1* *kb)* vector was used to drive expression in sensory neurons using the 3.1 kb upstream promotor region for the *ngn1* gene^46^. For expression of membrane-tethered eGFP, we used the *pMe-eGFP*^*−*^*CAAX* and *p3e-PA*. Multiple site gateway recombination of these vectors was accomplished using linearized *pDestTol2CG2*.

### PA-Rac1 expression

A *tol2–4xnr UAS-PA Rac1-mcherry* plasmid was diluted to a working solution of 75 ng/µl. This working solution was further diluted to 12 ng/µl in water along with 25 ng/µl of *transposase* mRNA. This injection solution was injected into single-cell *Tg(sox10:gal4); Tg(uas:lifeact-GFP)* embryos. At 48 h, injected embryos were screened for *mcherry* expression via confocal microscopy. *mcherry*^*+*^ DRG were imaged for 24 h either with or without exposure to 445 nm light. Mature PA-Rac1 photoactivates upon exposure to 445 nm light. *tol2–4xnr UAS-PA Rac1-mcherry* was a gift from Anna Huttenlocher (Addgene plasmid #41878).

### Tracing of growth cones

ImageJ plugin MTrackJ was used for tracking individual growth cones. Movies were converted to Tiff-OME and each time point within a 24-h movie was manually tracked. Individual scientists who performed the tracking were blind to the hypothesis and predicted outcome of the given experiment. Accuracy of the tracks was verified by a second scientist.

### Axon entry measurements

Y-orthogonal images of the glia limitans and navigating axons were generated by digitally rotating images 90° using Slidebook software. These images were converted to Tiff-OME files and the *y*- and *z*-planes of the image were traced in ImageJ. For the quantifications, the *y*-plane was established as the edge of the glia limitans, and the *z*-plane was horizontal plane of the *y*-orthogonal image through the growth cone as determined by the *Tg(sox10:lifeact-GFP)* signal. From these tracings, the intensities of the *Tg(gfap:nsfb-mcherry)* and *Tg(sox10:lifeact-GFP)* signals were individually plotted over the distance of the tracing of the plane by the plot profile analysis in ImageJ.

### Quantification and statistical analysis

Slidebook software was used to generate composite *z*-images for the cell and nerve counts. Individual *z*-images were sequentially observed to confirm composite accuracy. All graphically presented data unless otherwise noted represent the mean of the analyzed data. Growth cone tracking was performed using the MTrackJ plugin for ImageJ, and all distances were collected using this software. Intensity values were collected with the MTrackJ plugin for ImageJ and normalized using the background intensity. The period before entry was defined as the 20 time points preceding the growth cone’s approach to the DREZ, the period of entry was defined as the 20 time points following the growth cone’s approach to the DREZ, and the period after entry was defined as the next 20 time points. For each of these measurements, the DREZ was defined as the location at which the pioneer axon entered or would enter the spinal cord. An axon was considered to have entered the spinal cord if its axon bifurcated after approaching the DREZ. Independent verification of this entry was then completed by rotating the images 90 degrees to visualize a cross-section. GraphPad Prism software was used to determine statistical analysis. Supplementary Table 1 represents the statistical parameters used in all figure panels.

### Reporting summary

Further information on experimental design is available in the Nature Research Reporting Summary linked to this article.

## Supplementary information

Supplementary Information

Description of Additional Supplementary Information

Supplemental Movie 1

Supplemental Movie 2

Supplemental Movie 3

Supplemental Movie 4

Supplemental Movie 5

Supplemental Movie 6

Supplemental Movie 7

Supplemental Movie 8

Reporting Summary

## Data Availability

All data generated in this manuscript are present in the figures and supplemental figures.

## References

[1] Raible DW, Ungos JM (2006). Specification of sensory neuron cell fate from the neural crest. Adv. Exp. Med. Biol..

[2] Julius D (2001). Molecular mechanisms of nociception. Nature.

[3] Basbaum AI, Bautista DM, Scherrer G, Julius D (2009). Cellular and molecular mechanisms of pain. Cell.

[4] Jessen KR, Mirsky R (1997). Embryonic Schwann cell development: the biology of Schwann cell precursors and early Schwann cells. J. Anat..

[5] Sharma K, Korade Z, Frank E (1995). Late-migrating neuroepithelial cells from the spinal cord differentiate into sensory ganglion cells and melanocytes. Neuron.

[6] Smith CJ (2017). TNFa/TNFR2 signaling is required for glial ensheathment at the dorsal root entry zone. PLoS Genet..

[7] Golding JP, Cohen J (1997). Border controls at the mammalian spinal cord: late-surviving neural crest boundary cap cells at dorsal root entry sites may regulate sensory afferent ingrowth and entry zone morphogenesis. Mol. Cell. Neurosci..

[8] Ramon y Cajal, S. *Histology of the Nervous System of Man and Vertebrates* (Oxford University Press, New York, 1911).

[9] Murphy DA, Courtneidge SA (2011). The ‘ins’ and ‘outs’ of podosomes and invadopodia: characteristics, formation and function. Nat. Rev. Mol. Cell Biol..

[10] Santiago-Medina M, Gregus KA, Nichol RH, O’Toole SM, Gomez TM (2015). Regulation of ECM degradation and axon guidance by growth cone invadosomes. Development.

[11] Nichols EL, Green LA, Smith CJ (2018). Ensheathing cells utilize dynamic tiling of neuronal somas in development and injury as early as neuronal differentiation. Neural Dev..

[12] McGraw HF, Nechiporuk A, Raible DW (2008). Zebrafish dorsal root ganglia neural precursor cells adopt a glial fate in the absence of neurogenin1. J. Neurosci..

[13] George D, Ahrens P, Lambert S (2018). Satellite glial cells represent a population of developmentally arrested Schwann cells. Glia.

[14] Kim J, Lo L, Dormand E, Anderson DJ (2003). SOX10 maintains multipotency and inhibits neuronal differentiation of neural crest stem cells. Neuron.

[15] Hines JH, Ravanelli AM, Schwindt R, Scott EK, Appel B (2015). Neuronal activity biases axon selection for myelination in vivo. Nat. Neurosci..

[16] Heap LA, Goh CC, Kassahn KS, Scott EK (2013). Cerebellar output in zebrafish: an analysis of spatial patterns and topography in eurydendroid cell projections. Front. Neural Circuits.

[17] McGraw H, Snelson CD, Prendergast A, Suli A, Raible DW (2012). Postembryonic neuronal addition in Zebrafish dorsal root ganglia is regulated by Notch signaling. Neural Dev..

[18] Green L, Smith CJ (2018). Single-cell photoconversion in living intact zebrafish. J. Vis. Exp..

[19] Kucenas S (2008). CNS-derived glia ensheath peripheral nerves and mediate motor root development. Nat. Neurosci..

[20] Bernardos RL, Raymond PA (2006). GFAP transgenic zebrafish. Gene. Expr. Patterns.

[21] Ramon y Cajal, S. *Recollections of My Life* (MIT Press, Cambridge, 1937).

[22] Erturk A, Hellal F, Enes J, Bradke F (2007). Disorganized microtubules underlie the formation of retraction bulbs and the failure of axonal regeneration. J. Neurosci..

[23] Schlatter MC, Buhusi M, Wright AG, Maness PF (2008). CHL1 promotes Sema3A-induced growth cone collapse and neurite elaboration through a motif required for recruitment of ERM proteins to the plasma membrane. J. Neurochem..

[24] Helker CSM (2013). The zebrafish common cardinal veins develop by a novel mechanism: lumen ensheathment. Development.

[25] Johnson K (2016). Gfap-positive radial glial cells are an essential progenitor population for later-born neurons and glia in the zebrafish spinal cord. Glia.

[26] Smith CJ, Johnson K, Welsh TG, Barresi MJF, Kucenas S (2016). Radial glia inhibit peripheral glial infiltration into the spinal cord at motor exit point transition zones. Glia.

[27] Murphy DA (2011). A Src-Tks5 pathway is required for neural crest cell migration during embryonic development. PLoS One.

[28] Quintavalle M, Elia L, Price JH, Heynen-Genel S, Courtneidge SA (2011). A cell-based high-content screening assay reveals activators and inhibitors of cancer cell invasion. Sci. Signal..

[29] Blake RA (2000). SU6656, a selective src family kinase inhibitor, used to probe growth factor signaling. Mol. Cell. Biol..

[30] Wauson EM, Guerra ML, Barylko B, Albanesi JP, Cobb MH (2013). Off-target effects of MEK inhibitors. Biochemistry.

[31] Herzog C, Haun RS, Ludwig A, Shah SV, Kaushal GP (2014). ADAM10 is the major sheddase responsible for the release of membrane-associated meprin A. J. Biol. Chem..

[32] Villerbu N, Gaben AM, Redeuilh G, Mester J (2002). Cellular effects of purvalanol A: a specific inhibitor of cyclin-dependent kinase activities. Int. J. Cancer.

[33] Moshfegh Y, Bravo-Cordero JJ, Miskolci V, Condeelis J, Hodgson L (2014). A Trio–Rac1–Pak1 signalling axis drives invadopodia disassembly. Nat. Cell Biol..

[34] Hoshino D (2013). Exosome secretion is enhanced by invadopodia and drives invasive behavior. Cell Rep..

[35] Coulpier F (2009). Novel features of boundary cap cells revealed by the analysis of newly identified molecular markers. Glia.

[36] Niederländer C, Lumsden A (1996). Late emigrating neural crest cells migrate specifically to the exit points of cranial branchiomotor nerves. Development.

[37] Maro GS (2004). Neural crest boundary cap cells constitute a source of neuronal and glial cells of the PNS. Nat. Neurosci..

[38] Tosney KW, Landmesser LT (1985). Specificity of early motoneuron growth cone outgrowth in the chick embryo. J. Neurosci..

[39] Gomez TM, Spitzer NC (1999). In vivo regulation of axon extension and pathfinding by growth-cone calcium transients. Nature.

[40] Dumontier M, Höcht P, Mintert U, Faix J (2000). Rac1 GTPases control filopodia formation, cell motility, endocytosis, cytokinesis and development in Dictyostelium. J. Cell. Sci..

[41] Buck KB, Zheng JQ (2002). Growth cone turning induced by direct local modification of microtubule dynamics. J. Neurosci..

[42] Hagedorn EJ (2013). The netrin receptor DCC focuses invadopodia-driven basement membrane transmigration in vivo. J. Cell Biol..

[43] Hagedorn EJ (2009). Integrin acts upstream of netrin signaling to regulate formation of the anchor cell’s invasive membrane in C. elegans. Dev. Cell.

[44] Ziel JW, Hagedorn EJ, Audhya A, Sherwood DR (2009). UNC-6 (netrin) orients the invasive membrane of the anchor cell in C. elegans. Nat. Cell Biol..

[45] Marjoram L (2015). Epigenetic control of intestinal barrier function and inflammation in zebrafish. Proc. Natl. Acad. Sci. USA.

[46] Andersen EF, Asuri NS, Halloran MC (2011). In vivo imaging of cell behaviors and F-actin reveals LIM-HD transcription factor regulation of peripheral versus central sensory axon development. Neural Dev..

[47] Kimmel CB, Ballard WW, Kimmel SR, Ullmann B, Schilling TF (1995). Stages of embryonic development of the zebrafish. Dev. Dyn..

[48] Kirby BB (2006). In vivo time-lapse imaging shows dynamic oligodendrocyte progenitor behavior during zebrafish development. Nat. Neurosci..

[49] Smith CJ, Morris AD, Welsh TG, Kucenas S (2014). Contact-mediated inhibition between oligodendrocyte progenitor cells and motor exit point glia establishes the spinal cord transition zone. PLoS Biol..

[50] Lisse TS (2016). Paclitaxel-induced epithelial damage and ectopic MMP-13 expression promotes neurotoxicity in zebrafish. Proc. Natl. Acad. Sci. USA.

[51] Kwan KM (2007). The Tol2kit: a multisite gateway-based construction kit forTol2 transposon transgenesis constructs. Dev. Dyn..

