# Supplementary material for: Pioneer axons employ Cajal’s battering ram to enter the spinal cord
Source: Nat Commun. 2019 Feb 4;10:562. doi: 10.1038/s41467-019-08421-9 (PMC6362287; doi:10.1038/s41467-019-08421-9)
Supplement: Supplementary file 1 — Supplementary Information [file 41467_2019_8421_MOESM1_ESM.pdf]

# Pioneer axons employ Cajal's battering ram to enter the spinal cord

Nichols and Smith

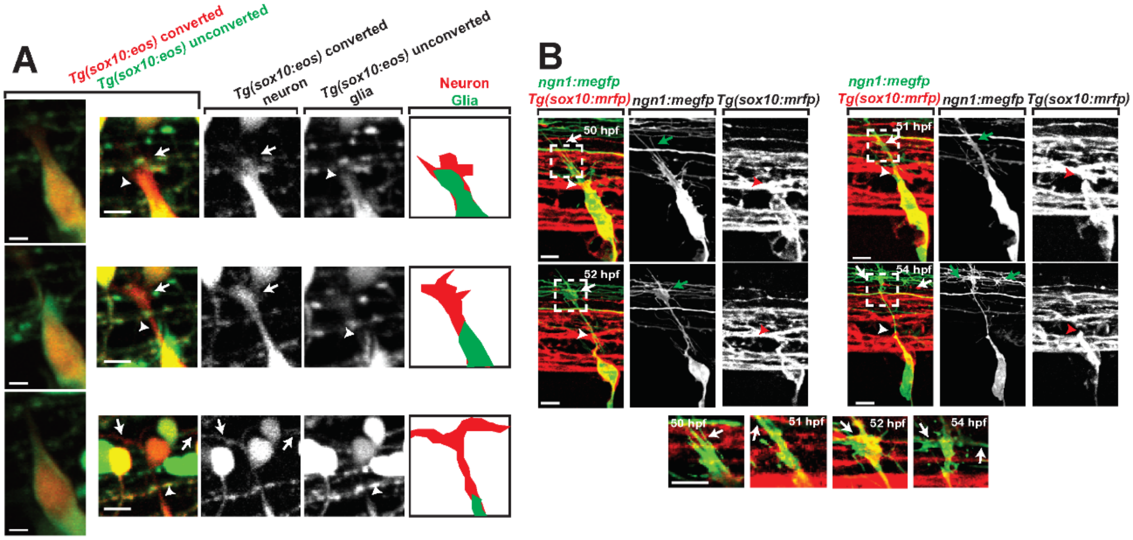


**Supplementary Figure 1. Glial projections do not extend with the pioneer axon to the DREZ, related to Figure 1.**

(A). Confocal z-projection frames from a 24-hr timelapse starting at 48 hpf of a *Tg(sox10:eos)* zebrafish with a photoconverted neuron. White arrow denotes pioneer axon growth cone. White arrowhead denotes trail-ing glial process. Images on the right show the growth cone contacting the DREZ with traced representations of the neuron (red) and glia (green) (B). Confocal z-projection frames of a 24-hr time-lapse starting at 48 hpf of a *Tg(sox10:mrfp)* with mosaic expression of *ngn1:megfp*. The pioneer axon leads the associated glia throughout its navigation before, during, and after DREZ entry. White and green arrows denotes growth cone, and white and red arrowheads denotes the trailing glia. Insets shown below represent the pioneer growth cone in single-planes. Scale bars denote 10 μm.

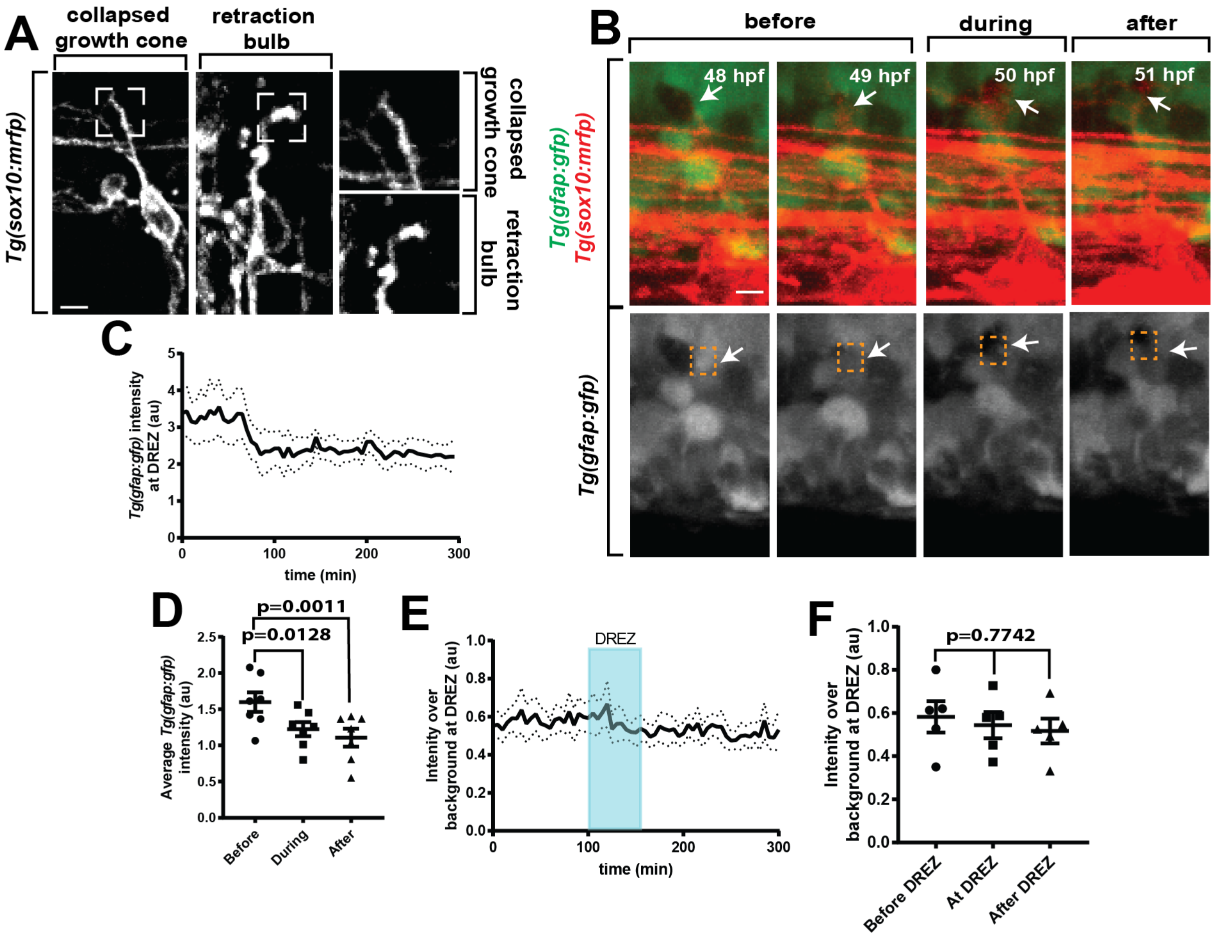


**Supplementary Figure 2. Axon entry corresponds with rearrangement of radial glia, related to Figure 2.**

(A). Confocal z-projection frames of *Tg(sox10:mrfp)* animals showing the morphologies of a collapsed growth cone and a retraction bulb. (B). Confocal z-projection frames from a 24-hr timelapse starting at 48 hpf of *Tg(sox10:mrfp); Tg(gfap:gfp)* zebrafish showing navigation of the pioneer axon crossing the radial glia membrane at the DREZ. White arrows denote the area of glial rearrangement during axon entry. Orange dotted box denotes DREZ. (C). Intensity profile of *Tg(gfap:gfp)* at the DREZ, n=7. Arrowhead denotes intensity during period of growth cone stalling at the DREZ. (D). *Tg(gfap:gfp)* intensity at the DREZ for before entry, during entry, and after entry. SEM is shown, n=7. (E). Quantification of *Tg(gfap:gfp)* intensity at the DREZ during failed entry into the spinal cord, SEM is shown, n=5 DREZ. (F). *Tg(gfap:gfp)* intensity at the DREZ during failed entry before DREZ navigation, during DREZ navigation, and after DREZ. SEM is shown, n=5. (D,F) use a one-way ANOVA and p-values in (D) are corrected for multiple comparisons. Scale bars denote 10 μm.

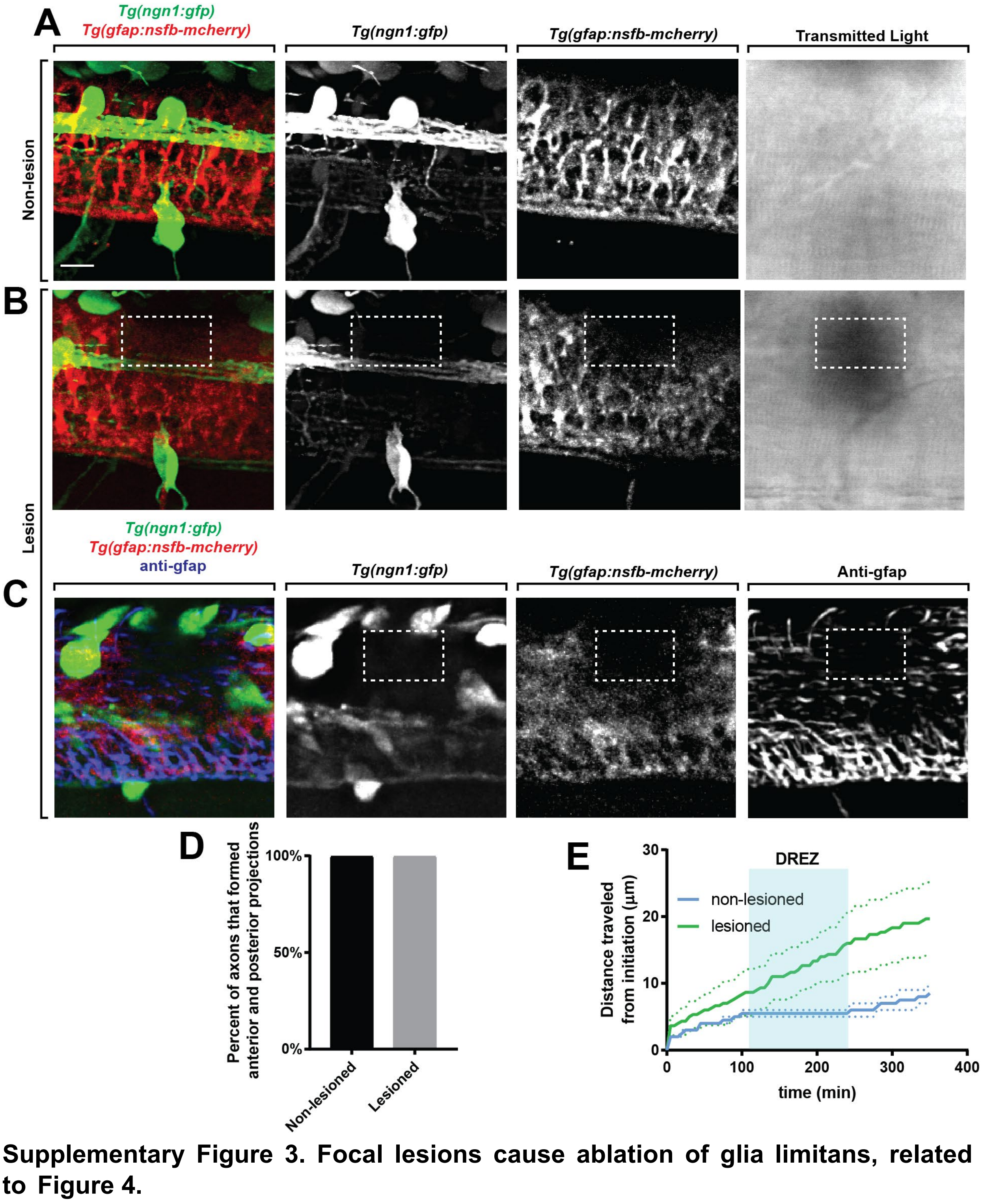


**Supplementary Figure 3. Focal lesions cause ablation of glia limitans, related to Figure 4.**

(A-B). Confocal images of a *Tg(ngn1:gfp); Tg(gfap:nsfb-mcherry)* zebrafish with (B) and without (A) a DREZ lesion. Images taken of the DREZ using transmitted light are also included. Dashed white box denotes lesion. (C). Confocal images of a *Tg(ngn1:gfp); Tg(gfap:nsfb-mcherry)* zebrafish with a lesioned DREZ stained for GFAP. Dashed white

box denotes lesion. (D). Quantification of the percent of axons with a lesioned and non-lesioned DREZ that form anterior and posterior projections in the spinal cord. n=5 lesioned, n=5 non-lesioned. (E). Graph of distance traveled by axons with a lesioned (green) and non-lesioned (blue) DREZ. Shaded blue box denotes the area of the DREZ. Dotted lines denote SEM. Note: Lesioned axons travel further distances because of the absence of stalling at the DREZ. Scale bars denote 10 μm.

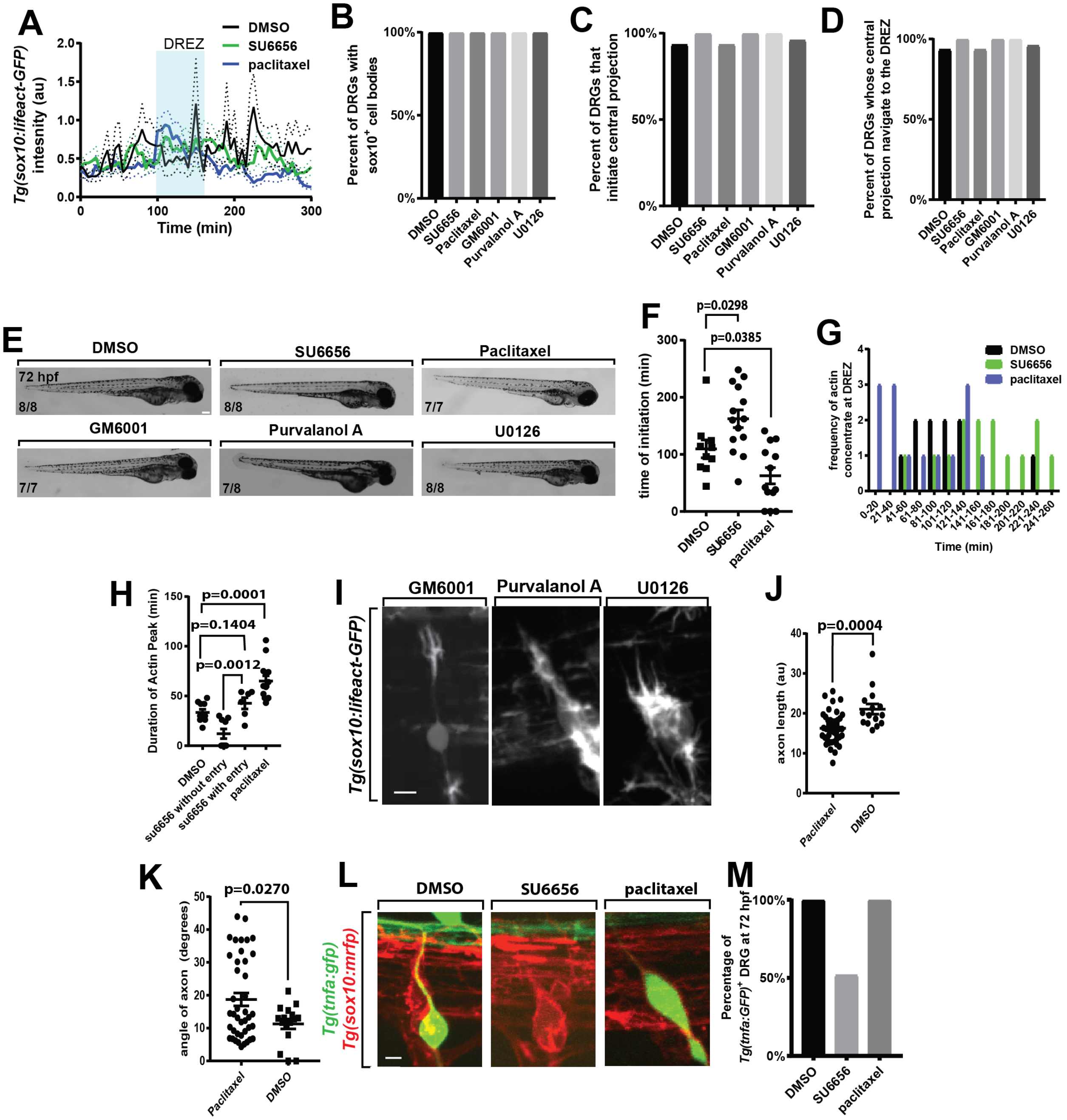

**Supplementary Figure 4. Pharmacological treatments impact pioneer axon DREZ entry, related to Figure 5.**

(A). Lifeact-GFP intensity tracings in the pioneer axon in animals treated with DMSO (black, n=14), SU6656 (green, n=20), and paclitaxel (blue, n=13). (B-D). Quantification of percent of DRG with Sox10+ cell bodies (B), that initiate a central projection (C), and that navigate to the DREZ (D) in animals treated with DMSO (n=14), SU6656 (n=20), paclitaxel (n=13), GM6001 (n=18), purvalanol A (n=18), and U0126 (n=20). (E). Images of embryo morphology at 72 hpf following treatment with DMSO, SU6656, paclitaxel, GM6001, purvalanol A, or U0126 starting at 36 hpf (F). Quantification of time of actin

concentrate initiation in animals treated with DMSO, SU6656, and paclitaxel. SEM is shown, n=14 nerves treated with SU6656, n=14 nerves treated with DMSO, n=13 nerves treated with paclitaxel. (G). Histogram of the time of actin concentrate formation in animals treated with DMSO (black), SU6656 (green), and paclitaxel (blue). n=14 nerves treated with SU6656, n=14 nerves treated with DMSO, n=13 nerves treated with paclitaxel. (H). Quantification of duration of actin concentrate in animals treated with DMSO, animals treated with paclitaxel, animals treated with SU6656 whose axons enter the spinal cord, and animals treated with SU6656 whose axons do not enter the spinal cord. SEM is shown, n=6 nerves treated with SU6656 that do not enter the spinal cord, n=9 nerves treated with SU6656 that enter the spinal cord, n=13 nerves treated with paclitaxel, n=14 nerves treated with DMSO. (I). Confocal z-projection images of a *Tg(sox10:lifeact-gfp)* animal treated at 36 hpf with GM6001, purvalanol A, and U0126. (J). Quantification of axon length in animals treated with paclitaxel or DMSO. Length measured from the point of axon bifurcation in the spinal cord to the cell soma. SEM is shown, n=42 DRG treated with paclitaxel, n=15 DRG treated with DMSO. Angle measured from the point of axon bifurcation in the spinal cord to the cell soma. SEM is shown, n=42 DRG treated with paclitaxel, n=15 DRG treated with DMSO. (K). Quantification of axon angle in animals treated with paclitaxel or DMSO. (L). Confocal z-projection frames taken at 72 hpf of *Tg(tnfa:gfp), Tg(sox10:mrfp)* zebrafish showing the DRG cell body and axon in animals treated with DMSO, SU6656, and paclitaxel. (M). Quantification of DRG expressing *Tg(tnfa:gfp)* at 72 hpf in animals treated with DMSO (n=60 DRG), SU6656 (n=118 DRG), and paclitaxel (n=68). SEM is shown. Scale bars denote 0.1 mm in (E) and 10 µm in (I,L). (F,H) use a Tukey's HSD and (J,K) use a Student's *t*-test.

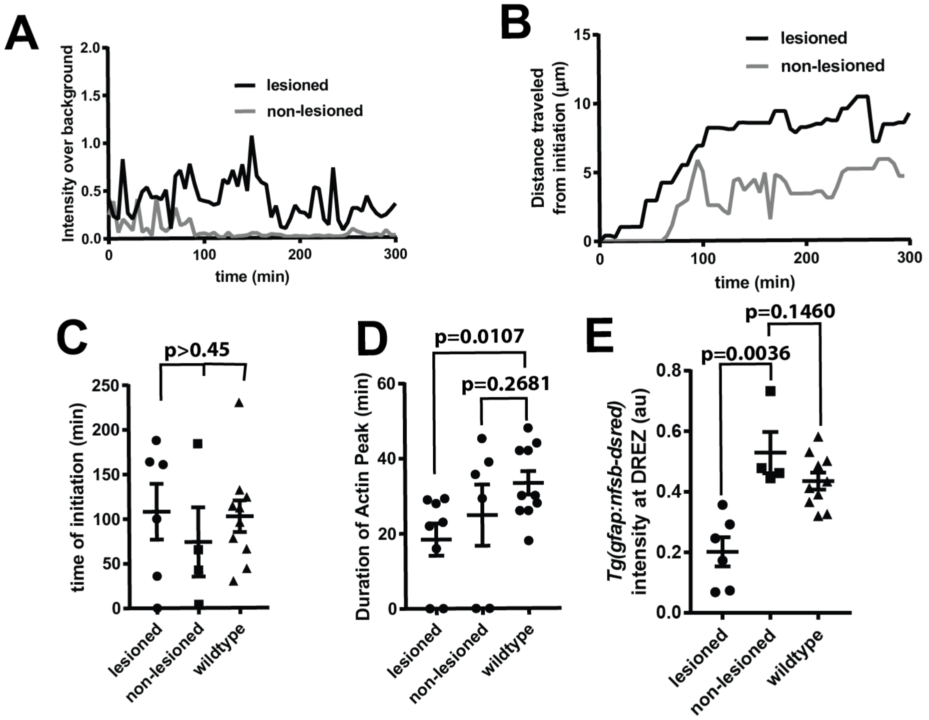


**Supplementary Figure 5. DREZ lesions and SU6656 treatment disrupt actin-rich invasion component formation and dynamics, related to Figure 7.**

(A). Representative intensity profile of Lifeact-GFP expression at the growth cone in animals treated with SU6656 and a lesioned DREZ (black) and animals treated with SU6656 and without a lesioned DREZ (gray). (B). Representative quantification of the distance traveled by the growth cone from the point of initiation through approach to the DREZ and movement anterior in the spinal cord in animals treated with SU6656 and a lesioned DREZ (black) and animals treated with SU6656 without a lesioned DREZ (gray). (C). Quantification of time of initiation of an actin concentrate in wildtype animals (n=10 DRG), lesioned and SU6656 treated animals (n=6 DRG), and non-lesioned and SU6656 treated animals (n=4 DRG). SEM is shown. (D). Quantification of duration of an actin concentrate in wildtype animals (n=10 DRG), lesioned and SU6656 treated animals (n=6 DRG), and non-lesioned animals (n=4 DRG). (E). Quantification of relative intensity of *Tg(gfap:nsfb-mcherry)* at the DREZ in wildtype animals (n=10 DRG), animals treated with SU6656 and a DREZ lesion (n=6 DRG), and animals treated with SU6656 without a DREZ lesion (n=4 DRG) animals. SEM is shown. (C-E) use a Tukey's HSD.

| Figure number | Raw Number | n value: Number of Animals Represented in Panel | n value: Number of DRG Represented in Panel | n value: Total Number of Animals Assayed | n value: Total Number of DRG Assayed | p-value | Statistical test |
|---|---|---|---|---|---|---|---|
| 1A | | | | | | | |
| 1B | Soma-ensheathing glia: 14.46<br>Pioneer axon: 18.075 | 5<br>5 | 18<br>18 | 5<br>5 | 18<br>18 | | |
| 1C | | Pre-entry: 1<br>Post-entry: 1 | Pre-entry: 1<br>Post-entry: 1 | Pre-entry: 4<br>Post-entry: 3 | Pre-entry: 16<br>Post-entry: 9 | | |
| 1D | Ganglia: 2.92<br>DREZ: 0 | 7 | 25 | 7 | 25 | <0.0001 | Fisher's exact |
| 1E | | 1 | 1 | 4 | 12 | | |
| 1F | | 1 | 1 | 4 | 12 | | |
| 1G | | 1 | 1 | 4 | 9 | | |
| 1H | | 4 | 9 | 4 | 9 | 0.0076 | Paired Student's |
| 2A | | 1 | 1 | 5 | 13 | | |
| 2B | | 1 | 1 | 5 | 13 | | |
| 2C | | | | | | | |
| 2D | | 5 | 13 | 5 | 13 | | |
| 2E | | 5 | 13 | 5 | 13 | | |
| 2F | | 4 | 4 | 4 | 16 | | |
| 2G | Before: 0.0361<br>During: 0.00476<br>After: 0.0209 | 4 | 4 | 4 | 16 | before vs. during: 0.0042<br>during vs. after: 0.0398 | Tukey's HSD |
| 3A | | 1 | 1 | 4 | 10 | | |
| 3B | | 1 | 1 | 4 | 10 | | |
| 3C | before: 18.591<br>during: 2.210<br>after: 24.519 | 6 | 6 | 4 | 10 | before vs. during: 0.0098<br>during vs. after: 0.0350 | Tukey's HSD |
| 3D | before: 18.591<br>during: 2.210<br>after: 24.519 | 6 | 6 | 4 | 10 | before vs. during: <0.0001<br>during vs. after: <0.0001 | Tukey's HSD |
| 3E | | 1 | 1 | 4 | 10 | | |
| 3F | | 1 | 1 | 4 | 11 | | |
| 3G | | 4 | 11 | 4 | 11 | | |
| 3H | | 4 | 11 | 4 | 11 | | |
| 3I | | 4 | 11 | 4 | 11 | | |
| 3J | | 1 | 1 | 4 | 11 | | |
| 4A | | 1 | 1 | 5 | 10 | | |
| 4B | | 1 | 1 | 5 | 9 | | |
| 4C | non-lesioned: 100<br>lesioned: 33.3 | non-lesioned: 5<br>lesioned: 5 | non-lesioned: 8<br>lesioned: 9 | non-lesioned: 5<br>lesioned: 5 | non-lesioned: 8<br>lesioned: 9 | | |
| 4D | | wildtype: 3<br>non-lesioned: 5<br>lesioned: 5 | wildtype: 10<br>non-lesioned: 8<br>lesioned: 9 | wildtype: 3<br>non-lesioned: 5<br>lesioned: 5 | wildtype: 12<br>non-lesioned: 8<br>lesioned: 9 | lesioned vs. non-lesioned: <0.0001<br>non-lesioned vs. wildtype: 0.1737 | Tukey's HSD |
| 4E | | 1 | 1 | Non-lesion: 5<br>Lesion: 5 | Non-lesion: 10<br>Lesion: 10 | | |
| 4F | | wildtype: 3<br>non-lesioned: 5<br>lesioned: 5 | wildtype: 10<br>non-lesioned: 8<br>lesioned: 9 | wildtype: 3<br>non-lesioned: 5<br>lesioned: 5 | wildtype: 12<br>non-lesioned: 8<br>lesioned: 9 | lesioned vs. non-lesioned: 0.0216<br>non-lesioned vs. wildtype: 0.0158 | Tukey's HSD |
| 4G | | wildtype: 3<br>non-lesioned: 5<br>lesioned: 5 | wildtype: 10<br>non-lesioned: 8<br>lesioned: 9 | wildtype: 3<br>non-lesioned: 5<br>lesioned: 5 | wildtype: 12<br>non-lesioned: 8<br>lesioned: 9 | lesioned vs. non-lesioned: 0.0012<br>non-lesioned vs. wildtype: 0.4819 | Tukey's HSD |
| 4H | | 1 | 1 | 5 | 9 | | |
| 4I | | 1 | 1 | 5 | 9 | | |
| 5A | | DMSO: 1<br>SU6656: 1<br>Paclitaxel: 1 | DMSO: 1<br>SU6656: 1<br>Paclitaxel: 1 | DMSO: 4<br>SU6656: 6<br>Paclitaxel: 4 | DMSO: 14<br>SU6656: 20<br>Paclitaxel: 13 | | |
| 5B | | DMSO: 1<br>SU6656: 1<br>Paclitaxel: 1 | DMSO: 1<br>SU6656: 1<br>Paclitaxel: 1 | DMSO: 4<br>SU6656: 6<br>Paclitaxel: 4 | DMSO: 14<br>SU6656: 20<br>Paclitaxel: 13 | | |
| 5C | DMSO: 92.9<br>SU6656: 55<br>Paclitaxel: 100<br>GM6001: 33.3<br>Purvalanol A: 50<br>U0126: 66.7 | DMSO: 4<br>SU6656: 6<br>Paclitaxel: 4<br>GM6001: 5<br>Purvalanol A: 4<br>U0126: 4 | DMSO: 14<br>SU6656: 20<br>Paclitaxel: 13<br>GM6001: 18<br>Purvalanol A: 18<br>U0126: 20 | DMSO: 4<br>SU6656: 6<br>Paclitaxel: 4<br>GM6001: 5<br>Purvalanol A: 4<br>U0126: 4 | DMSO: 14<br>SU6656: 20<br>Paclitaxel: 13<br>GM6001: 18<br>Purvalanol A: 18<br>U0126: 20 | | |
| 5D | | DMSO: 1<br>SU6656: 1<br>Paclitaxel: 1 | | DMSO: 48<br>SU6656: 86<br>Paclitaxel: 50 | | | |
| 5E | | DMSO: 48<br>SU6656: 86<br>Paclitaxel: 50 | | DMSO: 48<br>SU6656: 86<br>Paclitaxel: 50 | | | |
| 5F | DMSO: 32.8<br>SU6656: 4.5<br>Paclitaxel: 51.7 | DMSO: 48<br>SU6656: 86<br>Paclitaxel: 50 | | DMSO: 48<br>SU6656: 86<br>Paclitaxel: 50 | | <0.0001 | One-way ANOVA |
| 6A | | Photoactivated: 1<br>Non-photoactivated: 1 | Photoactivated: 1<br>Non-photoactivated: 1 | Photoactivated: 5<br>Non-photoactivated: 4 | Photoactivated: 9<br>Non-photoactivated: 10 | | |
| 6B | Photoacativated: 80<br>Non-photoactivated: 11.1 | Photoactivated: 5<br>Non-photoactivated: 4 | Photoactivated: 9<br>Non-photoactivated: 10 | Photoactivated: 5<br>Non-photoactivated: 4 | Photoactivated: 9<br>Non-photoactivated: 10 | | |
| 6C | | Photoactivated: 5<br>Non-photoactivated: 4 | Photoactivated: 9<br>Non-photoactivated: 10 | Photoactivated: 5<br>Non-photoactivated: 4 | Photoactivated: 9<br>Non-photoactivated: 10 | | |
| 6D | Photoacativated: 80<br>Non-photoactivated: 11.1 | Photoactivated: 5<br>Non-photoactivated: 4 | Photoactivated: 9<br>Non-photoactivated: 10 | Photoactivated: 5<br>Non-photoactivated: 4 | Photoactivated: 9<br>Non-photoactivated: 10 | | |
| 6E | | Photoactivated: 5<br>Non-photoactivated: 4 | Photoactivated: 9<br>Non-photoactivated: 10 | Photoactivated: 5<br>Non-photoactivated: 4 | Photoactivated: 9<br>Non-photoactivated: 10 | | |
| 6F | | 1 | 2 | 10 | 19 | | |
| 6G | | 1 | 1 | 7 | 15 | | |
| 7A | | Non-lesioned: 1<br>Lesioned: 1 | Non-lesioned: 1<br>Lesioned: 1 | Non-lesioned: 6<br>Lesioned: 6 | Non-lesioned: 9<br>Lesioned: 12 | | |
| 7B | SU6656 and lesion: 100<br>SU6656 and non-lesion: 55.5<br>SU6656: 55<br>DMSO: 92.9 | SU6656 and lesion: 6<br>SU6656 and non-lesion: 6<br>SU6656: 5<br>DMSO: 4 | SU6656 and lesion: 12<br>SU6656 and non-lesion: 9<br>SU6656: 20<br>DMSO: 14 | SU6656 and lesion: 6<br>SU6656 and non-lesion: 6<br>SU6656: 5<br>DMSO: 4 | SU6656 and lesion: 12<br>SU6656 and non-lesion: 9<br>SU6656: 20<br>DMSO: 14 | | |
| 7C | DMSO and non-lesion: 83<br>SU6656 and non-lesion: 32<br>SU6656 and lesion: 66 | DMSO and non-lesion: 6<br>SU6656 and non-lesion: 6<br>SU6656 and lesion: 6 | DMSO and non-lesion: 30<br>SU6656 and non-lesion: 30<br>SU6656 and lesion: 28 | DMSO and non-lesion: 6<br>SU6656 and non-lesion: 6<br>SU6656 and lesion: 6 | DMSO and non-lesion: 30<br>SU6656 and non-lesion: 30<br>SU6656 and lesion: 28 | | |
| 7D | | | | | | | |
| Supplemental Figures | | | | | | | |
| S1A | | 1 | 1 | 4 | 9 | | |
| S1B | | 1 | 1 | 6 | 12 | | |
| | | Collapsed growth cone: 1 | Collapsed growth cone: 1 | Collapsed growth cone: 5 | Collapsed growth cone: 13 | | |

| | | | | | | | |
|---|---|---|---|---|---|---|---|
| S2A | | Retraction bulb: 1 | Retraction bulb: 1 | Retraction bulb: 6 | Retraction bulb: 6 | | |
| S2B | | 1 | 1 | 3 | 7 | | |
| S2C | | 3 | 7 | 3 | 7 | | |
| S2D | Before: 1.59<br>During: 1.23<br>After: 1.11 | 3 | 7 | 3 | 7 | Before vs. during: 0.0128<br>Before vs. after: 0.0011 | Tukey's HSD |
| S2E | | 5 | 5 | 6 | 20 | | |
| S2F | Before: 0.582<br>During: 0.544<br>After: 0.517 | 3 | 5 | 6 | 20 | 0.7742 | One-way ANOVA |
| S3A | | 1 | 1 | 5 | 10 | | |
| S3B | | 1 | 1 | 5 | 10 | | |
| S3C | | 1 | 1 | 7 | 14 | | |
| S3D | Non-lesioned: 100<br>Lesioned: 100 | Non-lesioned: 5<br>Lesioned: 5 | Non-lesioned: 8<br>Lesioned: 9 | Non-lesioned: 5<br>Lesioned: 5 | Non-lesioned: 8<br>Lesioned: 9 | | |
| S3E | | Non-lesion: 2<br>Lesion: 3 | Non-lesion: 2<br>Lesion: 3 | Non-lesion: 5<br>Lesion: 5 | Non-lesion: 10<br>Lesion: 10 | | |
| S4A | | DMSO: 4<br>SU6656: 6<br>Paclitaxel: 4 | DMSO: 14<br>SU6656: 20<br>Paclitaxel: 13 | DMSO: 4<br>SU6656: 6<br>Paclitaxel: 4 | DMSO: 14<br>SU6656: 20<br>Paclitaxel: 13 | | |
| S4B | DMSO: 100<br>SU6656: 100<br>Paclitaxel: 100<br>GM6001: 100<br>Purvalanol A: 100<br>U0126: 100 | DMSO: 4<br>SU6656: 6<br>Paclitaxel: 4<br>GM6001: 5<br>Purvalanol A: 4<br>U0126: 4 | DMSO: 14<br>SU6656: 20<br>Paclitaxel: 13<br>GM6001: 18<br>Purvalanol A: 18<br>U0126: 20 | DMSO: 4<br>SU6656: 6<br>Paclitaxel: 4<br>GM6001: 5<br>Purvalanol A: 4<br>U0126: 4 | DMSO: 14<br>SU6656: 20<br>Paclitaxel: 13<br>GM6001: 18<br>Purvalanol A: 18<br>U0126: 20 | | |
| S4C | DMSO: 93.3<br>SU6656: 100<br>Paclitaxel: 93.3<br>GM6001: 100<br>Purvalanol A: 100<br>U0126: 95.8 | DMSO: 4<br>SU6656: 6<br>Paclitaxel: 4<br>GM6001: 5<br>Purvalanol A: 4<br>U0126: 4 | DMSO: 14<br>SU6656: 20<br>Paclitaxel: 13<br>GM6001: 18<br>Purvalanol A: 18<br>U0126: 20 | DMSO: 4<br>SU6656: 6<br>Paclitaxel: 4<br>GM6001: 5<br>Purvalanol A: 4<br>U0126: 4 | DMSO: 14<br>SU6656: 20<br>Paclitaxel: 13<br>GM6001: 18<br>Purvalanol A: 18<br>U0126: 20 | | |
| S4D | DMSO: 93.3<br>SU6656: 100<br>Paclitaxel: 93.3<br>GM6001: 100<br>Purvalanol A: 100<br>U0126: 95.8 | DMSO: 4<br>SU6656: 6<br>Paclitaxel: 4<br>GM6001: 5<br>Purvalanol A: 4<br>U0126: 4 | DMSO: 14<br>SU6656: 20<br>Paclitaxel: 13<br>GM6001: 18<br>Purvalanol A: 18<br>U0126: 20 | DMSO: 4<br>SU6656: 6<br>Paclitaxel: 4<br>GM6001: 5<br>Purvalanol A: 4<br>U0126: 4 | DMSO: 14<br>SU6656: 20<br>Paclitaxel: 13<br>GM6001: 18<br>Purvalanol A: 18<br>U0126: 20 | | |
| S4E | DMSO: 100<br>SU6656: 100<br>Paclitaxel: 100<br>GM6001: 100<br>Purvalanol A: 87.5<br>U0126: 100 | DMSO: 1<br>SU6656: 1<br>Paclitaxel: 1<br>GM6001: 1<br>Purvalanol A: 1<br>U0126: 1 | | DMSO: 8<br>SU6656: 8<br>Paclitaxel: 7<br>GM6001: 7<br>Purvalanol A: 8<br>U0126: 8 | | | |
| S4F | DMSO: 96.33<br>SU6656: 162.4<br>Paclitaxel: 62.69 | DMSO: 4<br>SU6656: 6<br>Paclitaxel: 4 | DMSO: 14<br>SU6656: 14<br>Paclitaxel: 13 | DMSO: 4<br>SU6656: 6<br>Paclitaxel: 4 | DMSO: 14<br>SU6656: 14<br>Paclitaxel: 13 | DMSO vs. SU6656: 0.0298<br>SU6656 vs. Paclitaxel: 0.0385 | Tukey's HSD |
| S4G | DMSO: 96.33<br>SU6656: 162.4<br>Paclitaxel: 62.69 | DMSO: 4<br>SU6656: 6<br>Paclitaxel: 4 | DMSO: 14<br>SU6656: 14<br>Paclitaxel: 13 | DMSO: 4<br>SU6656: 6<br>Paclitaxel: 4 | DMSO: 14<br>SU6656: 14<br>Paclitaxel: 13 | | |
| S4H | DMSO: 33.4<br>SU6656 without entry: 12<br>SU6656 with entry: 42.67<br>Paclitaxel: 65 | DMSO: 4<br>SU6656 without entry: 6<br>SU6656 with entry: 6<br>Paclitaxel: 4 | DMSO: 14<br>SU6656 without entry: 6<br>SU6656 with entry: 9<br>Paclitaxel: 13 | DMSO: 4<br>SU6656 without entry: 6<br>SU6656 with entry: 6<br>Paclitaxel: 4 | DMSO: 14<br>SU6656 without entry: 6<br>SU6656 with entry: 9<br>Paclitaxel: 13 | DMSO vs. paclitaxel: 0.0001<br>DMSO vs. SU6656 without entry: 0.1404<br>SU6656 without entry vs. SU6656 with entry: 0.0012 | Tukey's HSD |
| S4I | | GM6001: 1<br>Purvalanol A: 1<br>U0126: 1 | GM6001: 1<br>Purvalanol A: 1<br>U0126: 1 | GM6001: 5<br>Purvalanol A: 4<br>U0126: 4 | GM6001: 18<br>Purvalanol A: 18<br>U0126: 20 | | |
| S4J | DMSO: 21.06<br>Paclitaxel: 16.28 | DMSO: 4<br>Paclitaxel: 9 | DMSO: 15<br>Paclitaxel: 42 | DMSO: 4<br>Paclitaxel: 9 | DMSO: 15<br>Paclitaxel: 42 | 0.0004 | Student's t-test |
| S4K | DMSO: 11.28<br>Paclitaxel: 18.72 | DMSO: 4<br>Paclitaxel: 9 | DMSO: 15<br>Paclitaxel: 42 | DMSO: 4<br>Paclitaxel: 9 | DMSO: 15<br>Paclitaxel: 42 | 0.027 | Student's t-test |
| S4L | | DMSO: 1<br>SU6656: 1<br>Paclitaxel: 1 | DMSO: 1<br>SU6656: 1<br>Paclitaxel: 1 | DMSO: 15<br>SU6656: 30<br>Paclitaxel: 17 | DMSO: 60<br>SU6656: 118<br>Paclitaxel: 68 | | |
| S4M | DMSO: 100<br>SU6656: 51.7<br>Paclitaxel: 100 | DMSO: 15<br>SU6656: 30<br>Paclitaxel: 17 | DMSO: 60<br>SU6656: 118<br>Paclitaxel: 68 | DMSO: 15<br>SU6656: 30<br>Paclitaxel: 17 | DMSO: 60<br>SU6656: 118<br>Paclitaxel: 68 | | |
| S5A | | Non-lesioned: 1<br>Lesioned: 1 | Non-lesioned: 1<br>Lesioned: 1 | Non-lesioned: 6<br>Lesioned: 6 | Non-lesioned: 9<br>Lesioned: 12 | | |
| S5B | | Non-lesioned: 1<br>Lesioned: 1 | Non-lesioned: 1<br>Lesioned: 1 | Non-lesioned: 6<br>Lesioned: 6 | Non-lesioned: 9<br>Lesioned: 12 | | |
| S5C | Lesioned: 0.202<br>Non-lesioned: 0.530<br>Wildtype: 0.435 | Lesioned: 6<br>Non-lesioned: 4<br>Wildtype: 3 | Lesioned: 6<br>Non-lesioned: 4<br>Wildtype: 10 | Lesioned: 6<br>Non-lesioned: 6<br>Wildtype: 3 | Lesioned: 12<br>Non-lesioned: 9<br>Wildtype: 10 | 0.45 | One-way ANOVA |
| S5D | Lesioned: 0.202<br>Non-lesioned: 0.530<br>Wildtype: 0.435 | Lesioned: 6<br>Non-lesioned: 6<br>Wildtype: 3 | Lesioned: 8<br>Non-lesioned: 6<br>Wildtype: 10 | Lesioned: 6<br>Non-lesioned: 6<br>Wildtype: 3 | Lesioned: 12<br>Non-lesioned: 9<br>Wildtype: 10 | Lesioned vs. Wildtype: 0.0107<br>Non-lesioned vs. Wildtype: 0.2681 | Tukey's HSD |
| S5E | Lesioned: 0.202<br>Non-lesioned: 0.530<br>Wildtype: 0.435 | Lesioned: 6<br>Non-lesioned: 6<br>Wildtype: 3 | Lesioned: 6<br>Non-lesioned: 4<br>Wildtype: 10 | Lesioned: 6<br>Non-lesioned: 6<br>Wildtype: 3 | Lesioned: 12<br>Non-lesioned: 9<br>Wildtype: 10 | Lesioned vs. Non-lesioned: 0.0036<br>Non-lesioned vs. Wildtype: 0.1460 | Tukey's HSD |

**Supplementary Table 1. Summary of statistical analysis.**
