# Supplementary material for: Pioneer axons employ Cajal’s battering ram to enter the spinal cord
Source: Nat Commun. 2019 Feb 4;10:562. doi: 10.1038/s41467-019-08421-9 (PMC6362287; doi:10.1038/s41467-019-08421-9)
Supplement: Supplementary file 2 — Description of Additional Supplementary Information [file 41467_2019_8421_MOESM2_ESM.pdf]

Description of Additional Supplementary Files

File Name: Supplementary Movie 1
Description: **Pioneer axons stall at the DREZ to cross radial glia.** Excerpt from a 24-h time-lapse confocal movie of *Tg(gfap:gfp); Tg(sox10:mrfp)* animals from 48-72 hpf. (Left) In *Tg(sox10:mrfp)* labeled dorsal root ganglia, pioneer axons navigate dorsally to the DREZ, pause, then enter to form anterior and posterior projecting axons inside the spinal cord. Red circle denotes the navigating pioneer axon. (Right) In *Tg(gfap:gfp)* labeled radial glial that line the edge of the spinal cord, cells rearrange during axon entry to allow for entry to occur. Red circle denotes the location of the entering pioneer axon. Frames in the video were captured every 5 minutes and video plays at 10 frames per second. Supplements Figure 1D.

File Name: Supplementary Movie 2
Description: **Actin dynamically rearranges at the growth cone upon reaching the DREZ.** Excerpt from a 24-h time-lapse movie of *Tg(sox10:lifeact-gfp)* animals from 48-72 hpf. In this *Tg(sox10:lifeact-gfp)* labeled dorsal root ganglia, actin rearranges in the growth cone to form a concentrate upon reaching the DREZ. Red circle denotes the navigating pioneer axon. Frames in the video were captured every 5 minutes and video plays at 10 frames per second. Supplements Figure 3A.

File Name: Supplementary Movie 3
Description: **Actin rearrangement corresponds to the rearrangement of radial glia.** Excerpt from a 24-h time-lapse movie of *Tg(sox10:lifeact-gfp); Tg(gfap:nsfb-mcherry)* animals from 48-72 hpf. (Left). A *Tg(sox10:lifeact-gfp)* labeled dorsal root ganglia axon exhibits actin rearrangement at the DREZ upon reaching the spinal the DREZ. Red circle denotes the pioneer axon (Middle). In *Tg(gfap:nsfb-mcherry)* labeled radial glia, cells' rearrangement corresponds with the formation of the Lifeact-GFP concentrate at the growth cone. Red circle denotes the location of pioneer axon entry. (Right). Composite image of *Tg(gfap:nsfb-mcherry)* labeled radial glia (red) and *Tg(sox10:lifeact-gfp)* (green) labeled dorsal root ganglia. Frames in the video were captured every 5 minutes and video plays at 10 frames per second. Supplements Figure 3G.

File Name: Supplementary Movie 4
Description: **Actin concentrate forms at the radial glia boundary during pioneer axon entry.** Excerpt from a 24-h time-lapse movie of *Tg(sox10:lifeact-gfp)(green); Tg(gfap:nsfbmcherry)(red)* animals from 48-72 hpf. (Left). Y-orthogonal frames of pioneer axon entry showing the formation of a Lifeact-GFP (green) concentrate on the radial glia boundary (red), corresponding to the axon reaching the DREZ. (Top). Dorsal view of spinal cord showing axons enter the spinal cord. (Right). Lateral z-projections showing the navigation of the pioneer axon to the DREZ. Frames in the video were captured every 5 minutes and the video plays at 10 frames per second. Supplements Figure 3G.

File Name: Supplementary Movie 5
Description: **Actin concentrate is dispensable for entry during when the DREZ is lesioned.** Excerpt from a 24-h time-lapse movie of *Tg(sox10:lifeact-gfp); Tg(gfap:nsfbmcherry)* animals with a lesioned DREZ from 48-72 hpf. (Left). A dorsal root ganglia axon labeled with *Tg(sox10:lifeact-gfp)* navigates to the DREZ and fails to form an actin concentrate. However, the axon still enters the spinal cord and forms anterior and posterior projections. Red circle denotes the navigating pioneer axon (Middle). The radial glia membrane, labeled with

Tg(gfap:nsfb-mcherry), has been ablated using focal lesions, allowing for the axon to enter without forming an actin concentrate. (Right). Composite image of *Tg(gfap:nsfb-mcherry)* labeled radial glia (red) and Tg(sox10:lifeact-gfp) (green) labeled dorsal root ganglia. Frames in the video were captured every 5 minutes and video plays at 10 frames per second. Supplements Figure 4C.

File Name: Supplementary Movie 6
Description: **Treatment with SU6656 prevents actin-rich invasion component formation and axon entry.** Excerpt from a 24-h time-lapse movie of *Tg(sox10:lifeact-gfp)* animals from 48-72 hours and treated with SU6656 at 36 hpf. The pioneer axon approaches the DREZ normally but fails to form invasion components. As a result, the axon cannot enter the spinal cord and travels back toward its cell body. Red circle denotes the navigating pioneer axon. Frames in the video were captured every 5 minutes and video plays at 10 frames per second. Supplements Figure 5A.

File Name: Supplementary Movie 7
Description: **Treatment with paclitaxel induces actin-rich invasion component formation.** Excerpt from a 24-h time-lapse movie of *Tg(sox10:lifeact-gfp)* animals from 48-72 hours and treated with paclitaxel. The pioneer axon travels dorsally but initiates invasion component formation early causing spinal cord entry before reaching the DREZ. Red circle denotes the navigating pioneer axon. Frames in the video were captured every 5 minutes and video plays at 10 frames per second. Supplements Figure 5A.

File Name: Supplementary Movie 8
Description: **DREZ lesioning rescues axon entry caused by failed invasion component formation.** Excerpt from a 24-hour time-lapse movie of *Tg(sox10:lifeact-gfp); Tg(gfap:nsfb-mcherry)* animal treated with SU6656 with a lesioned DREZ from 48-72 hours. (Left). A dorsal root ganglia axon labeled with *Tg(sox10:lifeact-gfp)* navigates dorsally to the DREZ and enters the spinal cord. The axon fails to form invasion components but still enters the spinal cord due to the DREZ lesion. Red circle denotes navigating pioneer axon. (Middle). The radial glia membrane, labeled with *Tg(gfap:nsfb-mcherry)*, has been ablated using focal lesions, allowing for the axon to enter despite its inability to form invasion components. (Right). Composite image of *Tg(gfap:nsfb-mcherry)* labeled radial glia (red) and *Tg(sox10:lifeact-gfp)* (green) labeled dorsal root ganglia. Frames in the video were captured every 5 minutes and video plays at 10 frames per second. Supplements Figure 7A.
