# Supplementary material for: Pioneer axons employ Cajal’s battering ram to enter the spinal cord
Source: Nat Commun. 2019 Feb 4;10:562. doi: 10.1038/s41467-019-08421-9 (PMC6362287; doi:10.1038/s41467-019-08421-9)
Supplement: Supplementary file 11 — Reporting Summary [file 41467_2019_8421_MOESM11_ESM.pdf]

nature research

Corresponding author(s): Cody Smith

Last updated by author(s): Dec 13, 2018

Reporting Summary

Nature Research wishes to improve the reproducibility of the work that we publish. This form provides structure for consistency and transparency in reporting. For further information on Nature Research policies, see Authors & Referees and the Editorial Policy Checklist.

Statistics

For all statistical analyses, confirm that the following items are present in the figure legend, table legend, main text, or Methods section.

n/a	Confirmed	
☐	☒	The exact sample size (*n*) for each experimental group/condition, given as a discrete number and unit of measurement
☐	☒	A statement on whether measurements were taken from distinct samples or whether the same sample was measured repeatedly
☐	☒	The statistical test(s) used AND whether they are one- or two-sided<br>*Only common tests should be described solely by name; describe more complex techniques in the Methods section.*
☒	☐	A description of all covariates tested
☐	☒	A description of any assumptions or corrections, such as tests of normality and adjustment for multiple comparisons
☐	☒	A full description of the statistical parameters including central tendency (e.g. means) or other basic estimates (e.g. regression coefficient) AND variation (e.g. standard deviation) or associated estimates of uncertainty (e.g. confidence intervals)
☐	☒	For null hypothesis testing, the test statistic (e.g. *F*, *t*, *r*) with confidence intervals, effect sizes, degrees of freedom and *P* value noted<br>*Give P values as exact values whenever suitable.*
☒	☐	For Bayesian analysis, information on the choice of priors and Markov chain Monte Carlo settings
☒	☐	For hierarchical and complex designs, identification of the appropriate level for tests and full reporting of outcomes
☒	☐	Estimates of effect sizes (e.g. Cohen's *d*, Pearson's *r*), indicating how they were calculated

Our web collection on statistics for biologists contains articles on many of the points above.

Software and code

Policy information about availability of computer code

Data collection	All confocal microscopy images were acquired using a spinning disk confocal from 3i Technology. Slidebook software was used to generate composite z-images.
Data analysis	All image analysis was completed using ImageJ. The MTrackJ plugin was used for cell tracing measurements.

For manuscripts utilizing custom algorithms or software that are central to the research but not yet described in published literature, software must be made available to editors/reviewers. We strongly encourage code deposition in a community repository (e.g. GitHub). See the Nature Research guidelines for submitting code & software for further information.

Data

Policy information about availability of data

All manuscripts must include a data availability statement. This statement should provide the following information, where applicable:

- Accession codes, unique identifiers, or web links for publicly available datasets
- A list of figures that have associated raw data
- A description of any restrictions on data availability

All data generated in this manuscript are present in the figures and supplemental figures. Data is available from the corresponding author upon request.

Field-specific reporting

Please select the one below that is the best fit for your research. If you are not sure, read the appropriate sections before making your selection.

☒ Life sciences ☐ Behavioural & social sciences ☐ Ecological, evolutionary & environmental sciences

Life sciences study design

All studies must disclose on these points even when the disclosure is negative.

Sample size	Sample sizes were determined based on data from previous studies.
Data exclusions	No data was excluded in this study.
Replication	Attempts at replicating these data were successful.
Randomization	Animals were sorted into experimental groups at random. A normal distribution was assumed for all statistical analyses.
Blinding	Quantification of data was performed by a scientist blinded to the hypotheses when possible.

Reporting for specific materials, systems and methods

We require information from authors about some types of materials, experimental systems and methods used in many studies. Here, indicate whether each material, system or method listed is relevant to your study. If you are not sure if a list item applies to your research, read the appropriate section before selecting a response.

Materials & experimental systems

n/a	Involved in the study	
☐	☒	Antibodies
☒	☐	Eukaryotic cell lines
☒	☐	Palaeontology
☐	☒	Animals and other organisms
☒	☐	Human research participants
☒	☐	Clinical data

Methods

n/a	Involved in the study	
☒	☐	ChIP-seq
☒	☐	Flow cytometry
☒	☐	MRI-based neuroimaging

Antibodies

Antibodies used	Zrf-1 (ZIRC, ZFIN ID: ZDB-ATB-081002-46), anti-Sox10 (Kucenas lab), anti-Cortactin (Sigma, catalog number: SAB4500766), pSrc (Y418) (Invitrogen, catalog number: 44-660G), Alexa Flour 594 goat anti-rabbit (Invitrogen, catalog number: A-11037), Alexa Flour 594 goat anti-mouse (Invitrogen, catalog number: R37121), and Alexa Fluor 647 goat anti-mouse (Invitrogen, catalog number: A-21235)
Validation	Validation statements can be found on the manufacturers' websites.

Animals and other organisms

Policy information about studies involving animals; ARRIVE guidelines recommended for reporting animal research

Laboratory animals	Zebrafish strains used were as follows: AB, Tg(ngn1:gfp), Tg(sox10:mrfp), Tg(gfap:gfp), Tg(sox10:gal4), Tg(uas:lifeact-gfp), Tg(gfap:nsfb-mcherry), Tg(tnfa:gfp), and Tg(sox10:eos), Tg(uas:mcherry). All animals were used from 2-4 days post fertilization.
Wild animals	No wild animals were used in this study.
Field-collected samples	No field-collected samples were used in this study.
Ethics oversight	Ethics oversight was performed by the University of Notre Dame Institutional Animal Care and Use Committee.

Note that full information on the approval of the study protocol must also be provided in the manuscript.
